# Research progress of cell membrane biomimetic nanoparticles for circulating tumor cells

**DOI:** 10.3389/fonc.2024.1389775

**Published:** 2024-04-30

**Authors:** Yingfeng Zhang, Jia Wang

**Affiliations:** Department of Gynecology and Obstetrics, University-Town Hospital of Chongqing Medical University, Chongqing, China

**Keywords:** biomimetic nanoparticles, cell membrane, circulating tumor cells, diagnosis, progress

## Abstract

Early detection of cancer is crucial to reducing fatalities and improving patient outcomes. Metastasis is the first stage of aggressive cancers, often occurring before primary lesions can be seen. It occurs when cancerous cells disseminate to distant, non-malignant organs through the bloodstream, known as circulating tumor cells (CTCs). CTCs, or cancer tumor cells, are valuable indicators for predicting treatment response, metastasis progression, and disease progression. However, they are primarily used for research due to challenges like heterogeneity, separation from blood, and lack of clinical validation. Only a few methods have been approved for clinical use. One area of research is the isolation and identification of CTCs, which could significantly impact early cancer detection and prognosis. Current technologies using whole-blood samples use size, immunoaffinity, and density approaches, along with positive and negative enrichment techniques. Surface modification of nanomaterials is important for effective cancer therapies because it improves their ability to target and reduces interactions with healthy tissues. Consequently, researchers have created biomimetic nanoparticles covered with cell membranes using functional, targeted, and biocompatible coating technology. Nanoparticles with membranes can target specific cells, stay in circulation for longer, and avoid immune responses, which makes them much better at capturing CTCs. This study examines the current opportunities and difficulties associated with using cell membrane–coated nanoparticles as a capture technique for CTCs. In addition, we examine potential future developments in light of the current obstacles and investigate areas that require further research to fully understand its growing clinical possibilities.

## 1 Introduction

**Figure 1:**
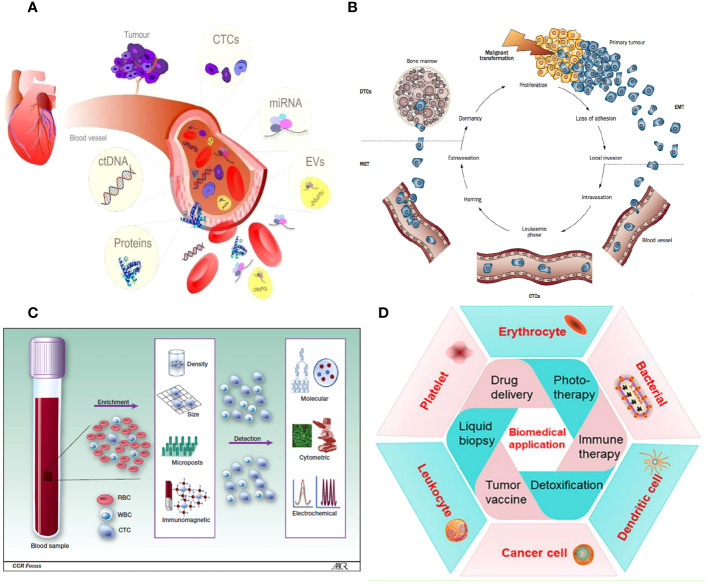
**(A)** Liquid biopsy. **(B)** CTCs are released from the main tumor and have a direct role in the process of metastasis, as described by Paget’s “seed and soil” hypothesis. **(C)** Isolation and identification of CTC. **(D)** A wide variety of cell membrane types have been extensively studied for their ability to encapsulate nanoparticles.

Cancer-related diseases are a major global health concern due to escalating mortality rates and limited treatment options. Early cancer detection and diagnosis are crucial for improving patient outcomes (1). Current diagnostic techniques, such as tissue biopsies, imaging techniques, and tumor markers, are inadequate for capturing all aspects of a patient’s cancer due to their inability to meet diagnostic criteria, sampling biases, and limited access to metastatic lesions. Histopathology is considered the most reliable method, but advanced imaging technologies like MRI and PET-CT can detect primary tumors and metastases. Liquid biopsy tests, which measure tumor components, have emerged as a recognized technique for molecular screening and early detection of cancers (**Figure 1A**) (2).

Circulating tumor cells (CTCs) are cancer cells that spread from the main tumor and survive in the bloodstream, forming new tumors. They undergo epithelial-to-mesenchymal transition (EMT) to invade surrounding tissue. However, the efficiency of this process is limited, and not all CTCs in the vascular system can form metastases (**Figure 1B**) (3). The presence of CTCs in the bloodstream could serve as an early sign of cancer metastasis, allowing non-invasive monitoring and early identification of aggressive forms of cancer. This could contribute to the diagnosis and management of cancer. Over the past decade, research has improved techniques for detecting, separating, isolating, and analyzing colorectal cancer cells using micro-fluidic systems and genomic analysis (**Figure 1C**). CTCs have demonstrated prognostic efficacy in predicting progression-free and overall survival in patients with metastatic prostate, breast, and colorectal cancer (4, 5). However, their scarcity makes it difficult to integrate them into routine clinical procedures. Advancements in precision medicine have prompted a thorough examination of CTCs.

Despite significant advancements in nanoengineering and biomaterial modification on nanoparticle (NP) surfaces, unforeseen material features can have adverse effects on the functionality of NPs in physiologically relevant systems (6, 7). In recent times, a range of nanoplatforms have been created for biomedical purposes owing to their excellent biocompatibility, substantial drug loading capacity, controlled drug release capability, and improved tumor penetrating ability (8). Nanoplatforms like dendrimers, nanogels, ultrasmall iron oxide NPs, carbon dots, and micelles have been made to help treat cancer, find cancer, or study the blood that cancer cells use. Nevertheless, these nanoplatforms encounter some shared challenges following intravenous delivery, including limited tumor targeting capability, brief circulation duration, and indiscriminate interactions with healthy tissues (9). Consequently, these obstacles impede their clinical application. An effective approach to tackling the challenges faced by nanomaterials is to incorporate functional or active, targeted molecules onto their surfaces (10). Even though different ligands, aptamers, or antibodies can be added to nanomaterial surfaces, they are not very useful because they are not very good at targeting and are quickly cleared away by the reticuloendothelial system (RES) (11).

Fortunately, biomimetic technology has emerged as a potential approach used to enhance the properties of nanomaterials, making them highly compatible with biological systems and reducing the likelihood of immune responses (12). Biomimetic NPs are a new type of NPs that combine the capabilities of biological materials with the adaptability of synthetic materials to successfully navigate and interact with intricate biological systems (13). The emergence of bio-inspired materials, which are derived from biological systems and can self-assemble or be combined with biocompatible synthetic materials, has ushered in the era of biomimetic technology (14). The application of NPs in biomedicine has been expanded through the use of biomimetic technologies, resulting in improved therapeutic and diagnostic outcomes, especially in the domain of pharmaceuticals. Biomimetic nanoconstructs are important in mitigating the rapid immunological response that occurs when administering different organic and inorganic NPs for medicinal conditions. Recently, there has been a combination of biomimetic and synthetic nanostructures in order to provide new features that can enhance biomedical applications (15, 16).

Cellular communication is essential in numerous biological processes, both normal and disease-related (17, 18). The use of natural cell membranes for coating NPs is a unique top-down strategy in nanotechnology’s rapid development (19). This developing method has led to the creation of several cell membrane–coated materials, which have acquired desired characteristics from the donor cells (14). Researchers covered NPs with erythrocyte membranes (EMs) in 2011 (20). This development then led to the creation of NPs covered with cell membranes (21, 22). This novel biomimetic NDDS integrates the distinct characteristics of natural biological units, including an extended duration of circulation. Consequently, it has the ability to deceive the host immune system and prevent the production of a protein layer, enhancing its compatibility with the body and extending its duration of stay (23, 24). Cell membrane bio-nanotechnology entails replicating the properties of a cell membrane by combining its natural properties with those of an artificial inner core material. To do this, separate the cell membrane from the cell and coat it with NPs that contain anti-tumor medications. This process significantly enhances the compatibility of the NPs with living organisms (25). Cell membrane–encapsulated NPs copy the features of cell membranes. This lets them stay in the bloodstream for longer, better identify antigens for more accurate targeting, interact more with cells, release medications more slowly, and be less harmful to living things. In addition, the cell membrane facilitates the delivery of the enclosed drug to the desired tissue and also serves to prevent early elimination, degradation, or premature release of the drug (26, 27). Therefore, membrane bio-nanotechnology surpasses the constraints of surface modification of traditional NPs in the field of biomedical applications (28, 29). Cell membranes from various sources can serve as viable sources for biological NPs (30). Biological NPs are frequently employed for the purpose of presenting antigens, delivering drugs to specific targets, and eliciting an immune response against tumors. The process by which membrane-coated NPs function involves targeted administration of therapeutic agents, immune system regulation, and precise therapeutic effects.

Moreover, NPs coated with a cell membrane can demonstrate intricate bio-interfacing capabilities. Various types of source cells are utilized for this objective, encompassing non-nucleated cells such as erythrocytes and platelets, prokaryotes, and eukaryotes like leukocytes. These cells are applied to NPs using methods such as co-extrusion, extrusion/sonication, freeze-thaw/sonication, extrusion/sonication and stirring, and others (30). In recent times, there has been a growing focus on cancer immunotherapy as a means to accomplish significant therapeutic advancements (31). Subsequently, the process of merging the inherent properties of a cell membrane, such as flexibility and biological traits, with a synthetic nanosystem that is functionally integrated has become significantly significant in several biomedical fields (32, 33). Currently, a wide variety of cell membrane types have been extensively studied for their ability to encapsulate NPs. Along with red blood cells (RBCs), platelets, and different kinds of white blood cells (WBCs; like T-lymphocytes, neutrophils, and macrophages), there are also cancer cells, stem cells, dendritic cells (DCs), natural killer (NK) cells, and membrane-derived structures such as exosomes, extracellular vesicles (EVs), and Outer membrane vesicles (OMVs). These diverse cell membrane types have the potential to be used in a range of applications, including drug delivery, phototherapy [such as photothermal therapy (PTT) and photodynamic therapy (PDT)], imaging, detoxification, cancer detection, immune modulation, and vaccination (**Figure 1D**).

**Figure 2:**
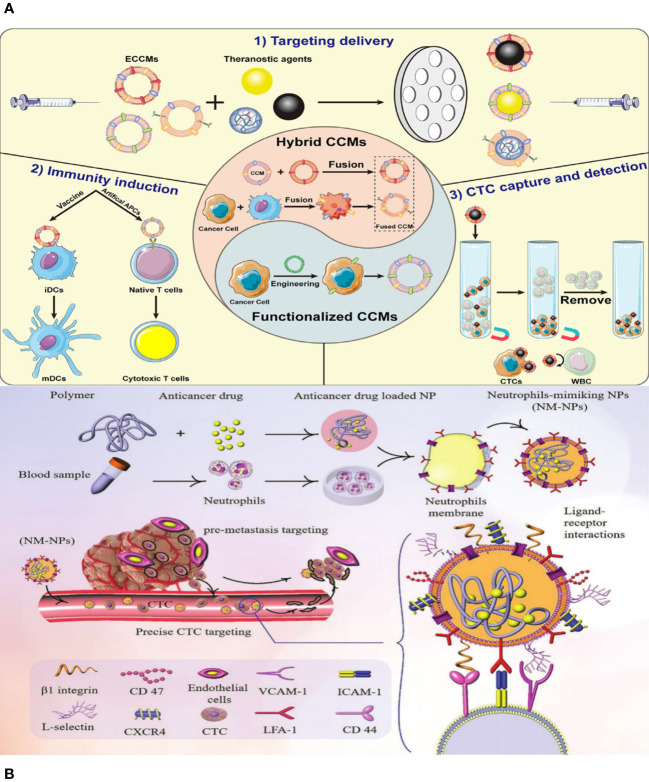
**(A)** Schematic illustration of the preparation of ethyl cellulose/chitosan microspheres (ECCMs) and their applications in cancer theranostics. **(B)** Schematic illustration of targeting CTCs and preventing them from forming new pre-metastatic clones through neutrophil-mimicking NPs (NM-NPs) used to deliver an anticancer drug.

Although bioinspired nanotechnology is thriving, monotypic cell membranes are unable to fulfil the varied and demanding requirements of biomedical applications. For instance, whereas the RBC membrane (RBCM) has excellent durability over extended circulation, it lacks the ability to specifically target the site of the disease (34). The cancer cell membrane can effectively target cells of the same type, but it has limited ability to evade the immune system due to the significant differences between laboratory-cultured tumor cells and those found in the body (35). Putting together different cell membranes that have the essential proteins and unique traits of their parent cells could make it possible for multifunctional biomimetic nanosystems to do complex tasks in living environments that are always changing (36). Researchers have found that hybrid membrane–based nanocarriers are more effective and safer than single-cell membrane–coated nanomaterials (**Figure 2A**) (37). Cancer cell membranes possess the ability to naturally and effectively target tumors by recognizing and attaching to specific membrane proteins on their surface, surpassing the capabilities of blood cell membranes. NPs that look like cell membranes can target cancer cells in living things and in the lab because the different membrane proteins on the surfaces of cancer cells are homologous and bind to each other well. Additionally, they can effectively avoid early detection by the immune system, penetrate the extracellular barrier, and evade elimination by the circulatory system.

In addition, the majority of recently developed methods primarily concentrate on active targeting in order to obtain more effective delivery of medicines, genes, and theranostics to specific locations of interest, as well as increased drug accumulation in the target cell(s) (38, 39). NPs and nanocarriers are changed by adding cell membranes and ligands like antibodies (like a-Herceptin and Rituxan), peptides (like a-RGD and b-NGR), nucleic acids, aptamers, folic acid (FA), b-CD19, a-Transferrin LHRH, a-Pegaptanib, a-Folate, and b-Galactose. The goal is to increase the specific binding of these particles to receptors that are overexpressed in the tumor microenvironment (TME). Various receptors have been identified for active targeting purposes, such as transferrin receptor (TfR), Nicotinic acetylcholine receptors (nAChRs), human epidermal growth factor receptor-2 (HER2), CD20, CD19 antigen, αvβ3 integrin, Aminopeptidase N, folate receptors (FAR), Asialoglyco-protein receptor, luteinizing hormone releasing hormone (LHRH) receptor, and Vascular Endothelial Growth Factor (VEGF) receptor (40). Active targeting increases the ability of medications to penetrate and accumulate in the TME by having a stronger attraction to the surface of target cells (**Figure 2B**).

This review discusses the biological features, enrichment and isolation technologies, and clinical applications of CTCs. It will also explore the different components and preparation techniques of biomimetic cell membranes, their advantages, and their biomedical applications. We further review current technologies for CTC detection and capture with cell membrane biomimetic NPs. A better understanding of CTC-based technologies and anticipated future developments will hopefully improve cancer treatment and diagnosis.

## 2 Cancers and CTCs

Significant advancements in understanding the biological basis of metastatic illness have occurred over the past two decades. Metastasis is the process by which cancer cells enter the bloodstream or lymphatic system and invade nearby tissues. While the tumor, node, and metastasis (TNM) staging method considers positive lymph node status as an indicator of advanced cancer, there is insufficient experimental evidence to confirm that cancer cells must pass through the lymphatic system before spreading to distant metastatic sites (15). The metastatic cascade begins when cancer cells invade the microenvironment and migrate through the endothelium barrier.

The circulatory or lymphatic systems carry away CTCs, which are tumor cells that have detached from the main humor. They are pointed out to serve as the foundation for metastasis, which involves invasive, metastasis-capable cell clones in the original tumor (41). CTCs can migrate to distant niches and develop into secondary lesions, with up to 1 × 10^6^ cancer cells released per gram of tumor tissue. However, their ability to form metastases is limited, with estimates around 0.01%.

Cancer cells undergo the EMT, allowing them to detach from the main tumor and enter the bloodstream. This process increases their metastasis potential and stem-like characteristics, triggering secondary tumor formation (42). The survival of cancer cells in the bloodstream is crucial, as they face challenges like hydrodynamic pressures, immune cell activities, anoikis, and sudden cellular oxygen levels. Cancer cells must quickly adapt to these factors (43). Evasion and reduced exposure to adverse bloodstream conditions can enhance the survival of CTCs. Elevated shear stress, fragmentation, and cell death can lead to the activation of pro-metastatic immune cells, facilitating the spread of surviving tumor cells (44). CTCs can undergo cell cycle arrest or produce anti-apoptotic proteins to avoid detection. Neutrophils promote CTC growth and survival while hindering the host’s adaptive and innate immune systems.

CTCs can extravasate into blood vessels when they interact with endothelial cells, forming visible metastatic lesions (45). Identifying CTCs in early-stage malignancies may not rule out curative treatment, but it may suggest aggressive malignancies that spread to other parts of the body. If there are no CTCs, then we may reduce the intensity of treatment. CTCs migrate and settle in new locations as disseminated tumor cells (DTCs), forming metastases. Anatomical factors and biological characteristics can affect their homing, with colorectal cancer involving a direct vascular link. Through chemotaxis, CTCs can migrate toward the bone marrow and attract granulocytes, forming cancerous growths in secondary locations (46). The surrounding milieu influences CTCs as they come to a halt and may enter dormancy. Cell types within organs provide safe havens for DTCs (47), and inflammation or osteoclast activity can trigger their activation and proliferation. Researchers have only recently acknowledged the significance of CTCs in cancer spread, despite technical difficulties in isolating them from circulating blood cells (48). New techniques have made it easier to use CTCs in cancer screening, therapy response, and prognosis.

## 3 Biological features of CTCs

Empirical data have substantiated the concept that neoplastic cells have the ability to disseminate, even in the first phases of tumor development (49). Characterizing the molecules of CTCs would enhance our understanding of the fundamental mechanisms involved in the spread of cancer, hence aiding in the early detection and prevention of metastasis.

### 3.1 Molecular characterization of CTCs

**Figure 3:**
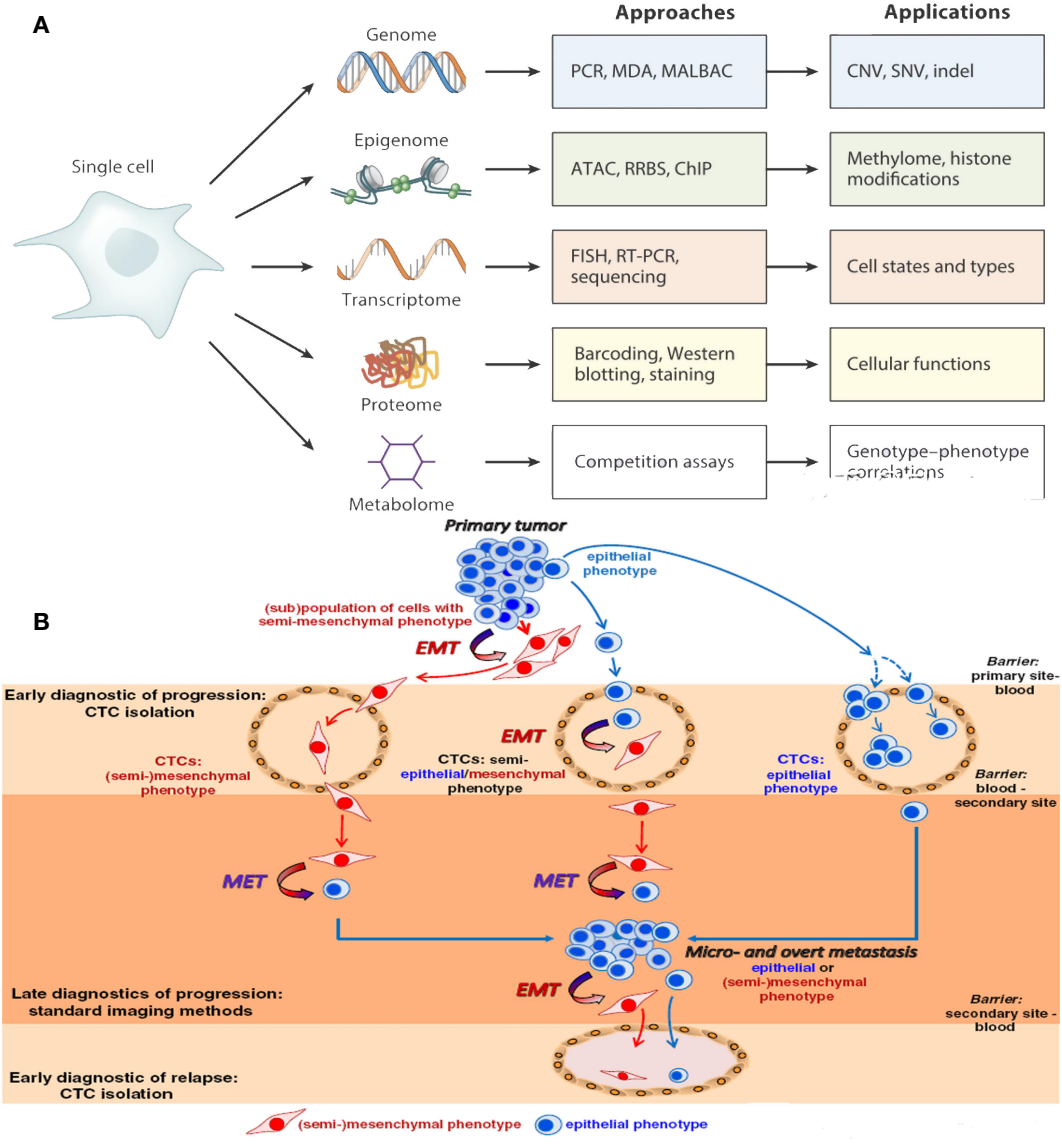
**(A)** An individual CTCs may now be examined to produce proteomic, transcriptomic, and epigenomic data. **(B)** Epithelial-to-mesenchymal transition of CTCs.

CTCs exhibit diverse physical characteristics and genetic makeup, contributing to the adaptive mechanisms necessary for cancer spread. Epithelial cell adhesion molecule (EpCAM), an epithelial marker for various types of cancer, serves as the primary marker to detect CTCs (50). However, looking only for EpCAM-positive CTCs may not provide a complete picture of the CTC population. Using both epithelial and mesenchymal cancer markers, as well as detection techniques that do not rely on markers, can improve the limited success of EpCAM-based technologies for isolating CTCs. EMT processes in cancer cells involve molecular changes, and EMT-related transcription factors are responsible for this. Various biomarkers, such as HER2, estrogen receptor, prostate-specific membrane antigen, folate receptor, and survival, have been identified as markers for CTCs in various types of cancer. CTC analysis should enable the customization of therapy choices and provide insights into the molecular mechanisms and transcriptional programs active in these cells (**Figure 3A**) (51).

### 3.2 Epithelial-to-mesenchymal transition of CTCs

EMT is a crucial process in cancer treatment, where epithelial tumor cells lose their intercellular adhesion and acquire mesenchymal and invasive characteristics. This process involves molecular, morphological, functional, and phenotypical alterations, converting polarized epithelial cells into mobile mesenchymal cells. This can create hybrid phenotypes that can survive in the bloodstream and adapt to different environments (52). Growth factors, transcription factors, and microRNAs initiate EMT during the initial phase of metastatic advancement. It amplifies the potential of tumors to form new tumors, spread to other parts of the body, resist radiation (53) and chemotherapy effects, and confer stem cell–like characteristics. EMT is a continuum, with three categories of cancer cell types: epithelial-like, mesenchymal-like, and a combination of both. CTCs with a hybrid E/M phenotype (54) can change between epithelial and mesenchymal states to fit their surroundings in the cancer. Targeting hybrid epithelial/mesenchymal CTCs may enhance patient survival rates, but further analysis is needed to fully characterize the phenotypic and molecular aspects of hybrid E/M CTCs (**Figure 3B**).

### 3.3 CTC clusters

Metastatic colonies contain various subclones with mutually beneficial relationships, suggesting cancer spreads through different groups of cells (55). Clusters of CTCs are found in 16%–75% of solid tumors, with groups having up to 100 times more potential to spread cancer than single CTCs. Clustering can occur among CTCs of the same kind or between different types of cells. Researchers have linked a higher number of clusters in patients’ blood to a worse progression-free survival (56). In cancer cases, invasive circulating tumor cells (iCTCs) can predict both the disease prognosis and the effectiveness of treatment.

### 3.4 Timing of CTC release

Circadian rhythms play a crucial role in cancer cell propagation, with chronotherapy aiming to optimize treatment times (57). However, we have only recently established the impact of circadian rhythm on CTC discharge and spread. Current methods for identifying CTCs assume no fluctuations, potentially constraining their practical application as a liquid biopsy analyte. New, time-controlled clinical studies are needed.

### 3.5 CTCS and the blood microenvironment

**Figure 4:**
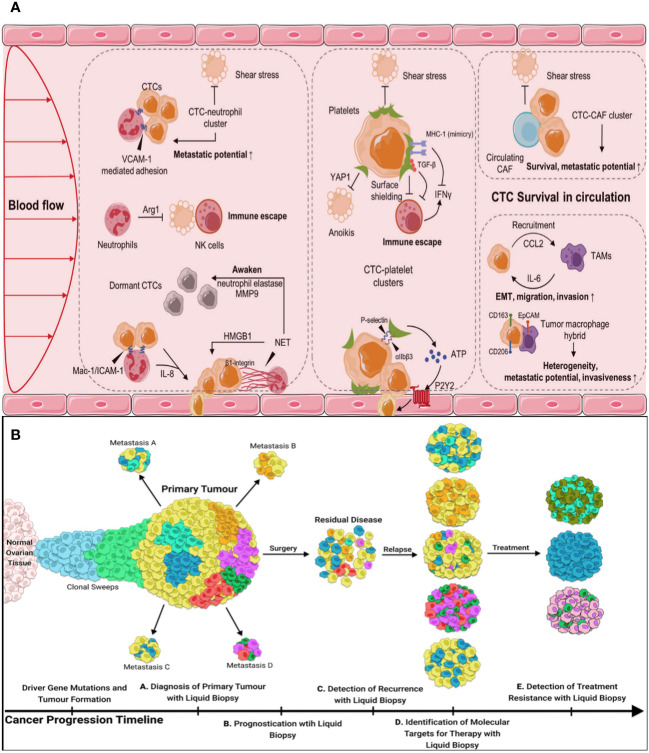
**(A)** CTCs in the blood microenvironment, and their interaction with neutrophils, platelets, CAFs, and TAMs. CAFs, cancer-associated fibroblasts, TAMs, tumor-associated macrophages. **(B)** The utility of liquid biopsy during different stages of tumor progression.

Harmful shear stress stops some CTCs in the bloodstream, or they undergo anoikis, a programmed cell death process that occurs when cells separate (58). Some CTCs hide from the immune system and improve their chances of survival by forming strong bonds with platelets, neutrophils, macrophages, myeloid-derived suppressor cells, or cancer-associated fibroblasts (CAFs) (**Figure 4A**) (59). Emerging research indicates that the interplay and regulation between CTCs and the hostile blood milieu are crucial for their attachment to endothelial cells, invasion of tissues, and the spread of tumors.

## 4 Clinical applications of CTCs

Several clinical trials have evaluated the capacity of CTCs to identify cancer by analyzing blood samples from individuals who have already been diagnosed with cancer. While the clinical guidelines have not yet incorporated the use of CTCs in clinical practice, they have included CTCs in the classification of cM0 tumors, which refers to tumors without obvious metastasis but with the presence of tumor cells in the blood. Numerous studies have anticipated the significant potential of CTCs in various clinical applications (**Figure 4B**).

### 4.1 Early diagnosis of cancer

CTC detection is a promising tool for early cancer diagnosis due to its non-invasive nature. However, there is ongoing debate over its practical usefulness. Although we see CTCs as indicators of metastatic activity, there is still debate over their spread in patients. Their limited presence and isolating process are the main challenges. Advanced tumors generate more CTCs, which migrate with metastatic clusters, facilitating their detection. Current clinical trials, like the PROLIPSY study for prostate cancer, breast cancer, non-small cell lung cancer (NSCLC), colorectal cancer (CRC), and pancreatic cancer (60), indicate the potential use of CTCs for early cancer detection.

### 4.2 Detecting recurrence and determining prognosis

Researchers are exploring the use of liquid biopsy to identify residual disease after primary debulking surgery, predict survival outcomes, and identify early disease recurrence. This information can help determine treatment strategies and clinical trials. CellSearch (61), the FDA-approved technique for clinical identification of CTCs, is considered a significant component for predicting outcomes (62). Studies show that higher CTC counts are associated with increased metastasis and cancer aggressiveness, lower survival rates, and a negative prognosis. The molecular characteristics of clinically characterized CTCs, including EMT and stemness, provide significant predictive value for prognosis.

### 4.3 Monitoring of the therapeutic response

Clinical trials have used CTCs to evaluate the effectiveness of cancer treatments (63), frequently in combination with imaging tests or serum biomarkers. CTCs have heightened sensitivity compared to imaging examinations and can reduce radiation exposure. Accurate identification of cancer subclones is crucial for determining the most suitable treatment and detecting resistance. CTCs can provide a more thorough examination of cancer differences and enable continuous tracking of tumor changes. However, there is limited use of them as a surrogate biomarker for cancer screening, therapy monitoring, and prognosis prediction (64). Quantification of CTCs in blood samples is used to inform therapy choices, but the presence of CTCs during cancer diagnosis or relapse is associated with prognosis (65). Single-cell omics techniques can provide valuable prognostic information on CTCs, and long-term follow-up data can help identify biomarkers linked to a condition’s progression.

## 5 Technologies for CTC enrichment and isolation

CTCs refer to cancer cells that are present in the bloodstream and are either released from a primary or metastatic solid tumor site by active intravazation or passive shedding. To incorporate CTCs as a liquid biopsy analyte in medical practice, capture technologies must be developed that are unbiased, efficient, fast, and cost-effective. These technologies should be able to consistently separate a sufficient quantity of CTCs. These capture methods must also be capable of working well with modern sequencing tools and functional assays in order to produce data that can be used to accurately categorize patients and make informed decisions about treatment. We can subject separated and preserved CTCs to a wide range of molecular and functional studies to explore the biology and weaknesses of metastatic cancer. CTCs are a unique type of analyte for liquid biopsies because they can represent aggressive subclones that are likely to spread. It is possible that phenotypic and molecular analysis could provide more valuable information compared to traditional tissue biopsies (sampling of random subclones) (66) or examination of other circulating substances, such as circulating tumor DNA (identification of dying subclones) (67). Nevertheless, this will require additional investigation. Utilizing less invasive blood draws could provide the opportunity for regular and continuous evaluation of the impact of clinical interventions and potentially facilitate the early identification of cancer or its reappearance (68). CTCs are considered an optimal source of biomarkers for immediate clinical applications and personalized therapy. However, trapping CTCs is difficult due to their infrequent occurrence. Therefore, it is crucial to develop effective methods for enriching CTCs in order to ensure consistent and reliable analysis and application in subsequent stages.

In recent years, numerous techniques have been suggested for capturing CTCs. CTC technologies typically employ three primary strategies (69): collection and enrichment, detection and identification, and release. The first step in capturing and enriching cells is for them to interact specifically with substances, either physically or through interactions between antibodies and antigens. CTC identification is the second way to find them. It includes a number of different technologies, such as fluorescence microscopy, fluorescence spectrophotometry, flow cytometry, surface-enhanced Raman scattering, and electrical impedance. The previous technique primarily utilized released CTCs for subsequent investigation, including genomes, transcriptomics, proteomics, and CTC culture.

### 5.1 Classic CTC–related technologies based on physical properties

**Figure 5:**
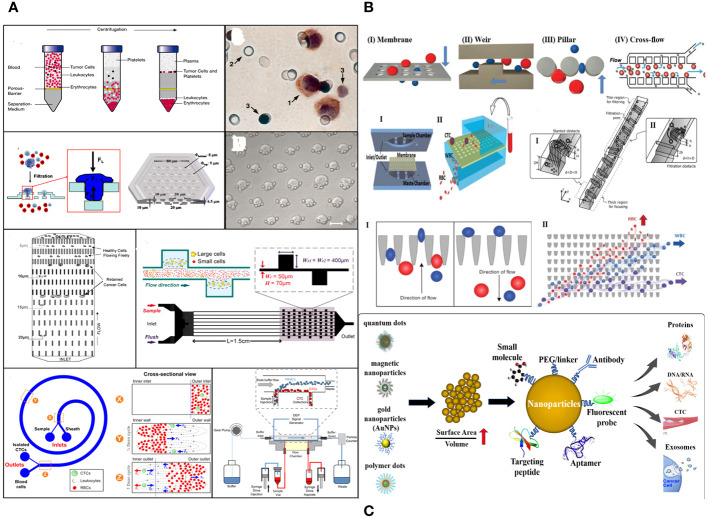
**(A)** Technologies for CTC enrichment based on physical properties. **(B)** Microfiltration technique for cell separation. **(C)** Nanotechnology improves cancer detection and diagnosis.

The physical separation enrichment technique for CTCs relies on disparities in size, density, deformability, and electrical characteristics between CTCs and blood cells (**Figure 5A**).

#### 5.1.1 Size-based

The presence or absence of antigens on the cell surface has no impact on size-based enrichment approaches, which are physical techniques. As a result, these methods avoid the uncertainty that arises from the varied antigen expression in CTCs (70). These techniques use the physical and mechanical distinctions between CTCs and other cells present in blood samples. Size-based approaches are physical techniques that separate CTCs based on their enlarged size, typically ranging from 9 μm to 19 μm. Size-based methods mostly employ membrane microfilters, whereas alternative microfluidic-based methods have also been documented (71). Size-based techniques offer several key benefits. Firstly, they allow for the isolation of label-free, unmodified, and viable cells. Secondly, these techniques are fast and simple to implement. Additionally, they exhibit a high capture efficiency and provide good enrichment, with a capture rate of 104 cells against leukocytes. Numerous studies (70) have supported this. Importantly, the cells obtained through size-based techniques can be utilized for subsequent downstream methods, such as next-generation sequencing (NGS). This enables researchers to extract more information from a single sample and enhance the detailed analysis of specific cancer types and cancer progression across different patients. Size-based approaches provide the advantage of faster enrichment time and lower cost compared to strategies that include biochemical changes, primarily because of the absence of costly labels (72). Nevertheless, a significant obstacle to isolating CTCs based on size is the potential interference by leukocytes, which have a similar size range to the lower end of CTCs (73).

#### 5.1.2 CTC membrane microfilters

Microfiltration techniques have been made possible by the utilization of microfabrication processes, which involve the construction of thin films made of polycarbonate. These films are designed to have precise nano- to micron-sized holes, which are created through surface bombardment (69). A flexible microspring array (FMSA) is a very efficient device that quickly separates CTCs based on their size and deformability (74). CTCs that are captured on the filter remain viable and can either be cultured within the device or removed using reverse flow. Live cells can undergo genetic examination, which yields diagnostic and prognostic information (75). A novel tandem FMSA (tFMSA) has recently been created for the purpose of selectively segregating cells according to variations in their sizes and deformability. A new type of microfilter called a separable bilayer (SB) microfilter has been created to capture CTCs based on their size (76). Clinomics has recently developed and commercialized a lab-on-a-disc platform that utilizes fluid-assisted separation technology. This technique enables the quick isolation of CTCs from whole blood, depending on their size, resulting in a reasonably high purity of isolated CTCs (77).

### 5.2 Classic CTC–related technologies based on biological properties

Immunoaffinity-based techniques for trapping CTCs were among the earliest methods discovered (69). These techniques rely on the use of particular antigens that are present on the surface of CTCs but not on other cells. To isolate CTCs from other blood cells, surface antigens are targeted using particular antibodies. When tumor cells are isolated, the method is categorized as positive enrichment. However, negative enrichment can also be employed, which involves labeling antigens that are not present on CTCs but are present on other blood cells. The antibodies used for capturing tumor cells are commonly attached to a surface within the device, which poses challenges in retrieving the captured cells following enrichment or recovering cells from magnetic particles (the immunomagnetic technique). In order to deplete unwanted cells, the CD45 antigen is typically used to specifically capture the normal cells that are present in the blood around the CTCs. Negative enrichment methods often provide lesser levels of purity compared to positive enrichment methods. However, the benefit of negative enrichment methods is that they allow for the isolation of label-free CTCs regardless of their unique antigen expression (78). CTCs are usually separated by using markers for epithelial (EpCAM) and mesenchymal (vimentin) cells to help with positive enrichment and CD45 to help get rid of unwanted WBCs, which is based on how antibodies and antigens interact (79). Researchers primarily use EpCAM-dependent approaches. The CellSearch system, which is the sole device approved by the FDA for clinical application, utilizes ferromagnetic beads coated with EpCAM antibodies to enhance the concentration of CK+/CD45−/DAPI+ CTCs and exclude CK−/CD45+/DAPI+ WBCs. Nevertheless, CTCs have a robust affinity for the equipment’s surface in antibody interaction-based techniques, rendering their release challenging. CanpatrolTM (80) is another example of the EpCAM-dependent method, which offers detailed analysis of the morphology, cytology, and genetics of individual CTCs.

The immunoaffinity-based methods for isolating CTCs have two primary drawbacks and difficulties. Firstly, the heterogeneity of CTCs can lead to the loss of certain subpopulations during the process of enrichment and capture. Secondly, the presence of CTCs bound to the surface of a device can create challenges in recovering these cells.

### 5.3 Recent CTC-related technologies: microfluidic-based and nanotechnology-based techniques

In recent times, the availability of microfluidic chips, nanomaterials, and NGS has provided researchers with a plethora of improved tools to drive advancements in CTC-related technologies. Researchers are working to improve many important parts of CTC technologies right now, such as yield, purity, enrichment ratio, throughput, viability, sensitivity, specificity, release rate, accessibility for further research, and how easy it is to use the equipment.

#### 5.3.1 Microfluidic immunocapture positive enrichment

To separate cells, microfluidic-based cell sorting methods use “intrinsic” forces, like fluid dynamic forces, instead of “extrinsic” forces, like magnetic, electric field, acoustic, and optical forces. These methods then identify and isolate target cells from a heterogeneous cell sample based on their distinct physical and biological properties (**Figure 5B**) (81). The CTC-chip is a silicon microfluidic technology that utilizes molecular marker–coated pins to capture CTCs. The CTC-chip (82) has the ability to isolate viable CTCs from whole blood without the need for pre-labeling or sample processing. This leads to enhanced cell activity and improved separation quality. In addition, the monolithic CTC-iChip (83) effectively removes WBCs and enables the analysis of CTCs that exhibit both epithelial and mesenchymal traits. These microfluidic chips have made it easier to find CTCs by improving the capture efficiency, viability, and depletion of WBCs. However, they are not widely used in clinical settings because they are expensive, take a long time to set up, and are bulky. They also cannot do single-cell molecular analysis. Altogether, low-cost, automated, and integrated microfluidic devices that make it easy to find CTCs and analyze cells have a lot of clinical value.

Glia et al. have successfully captured CTCs for the first time using an open biofunctionalized substrate that has the capacity to do multiplexing. The goal is reached by creating a new microfluidic probe (MFP) with radially staggered herringbone (HB) pieces that make microvortices. The newly developed device, known as the HB-MFP, is a microfluidic system that does not require channels. It consists of a capturing substrate located at the bottom and a fluidics delivery system positioned on top, which are physically separated from one another. In this research, different biorecognition ligands (in this case, different capture antibodies arranged in stripes) are added to the capture substrate. The fluid delivery system is then moved over the substrate in a 2D printing-like motion to facilitate the capture process. Targeting the EpCAM, prostate-specific membrane antigen (PSMA), and prostate-specific antigen (PSA) antigens of patients with prostate cancer in a single procedure makes the HB-MFP a good tool for collecting CTCs from their blood samples. The number of CTCs detected ranges from six CTCs mL-1 in patients with localized cancer to 280 CTCs mL-1 in patients with metastatic cancer. During the technique, CTC clusters containing 40–50 cells are effectively collected. The findings suggest that the use of multiplex profiles of CTCs can provide insights into specific cellular characteristics by analyzing the amounts of PSMA and PSA expression. The HB-MFP that has been created is user-friendly and durable, enabling efficient processing of a large number of samples and easy access to collected CTCs for additional analysis (84).

#### 5.3.2 Capture enhanced by nanomaterials

Nanotechnology-based techniques are increasingly becoming recognized as valuable instruments for early-stage illness detection and tracking cancer progression, as well as for *in vivo* imaging (**Figure 5C**) (85). This is due to advancements in nanomaterials. Nanomaterials possess a significant ratio of surface area to volume, enabling the isolation of CTCs with a high degree of specificity and the detection of CTCs with a high level of sensitivity. Adsorbing several targeted ligands onto the nanomaterials, which bind to particular molecules in cancer cells, achieves this. Currently, several nanomaterials have been documented in investigations for the detection of CTCs, such as magnetic NPs (86), gold NPs (87), and quantum dots (88). The use of tannic acid-functionalized magnetic NPs (89), CoFe2O4@Ag magnetic nanohybrids (90), and peptide-based magnetic NPs (86) has been shown to improve the ability to capture CTCs in patients with breast cancer. Peptide-based magnetic NPs can tell the difference between epithelial and mesenchymal CTC subgroups and allow research to be done on single cells. Magnetic NPs and integrated systems based on microfluidics (91) enhance the detection capabilities of these NPs. Moreover, the integration of a microfluidic system with gold NPs offers a broader spectrum of potential uses. While nanotechnology-based methods offer extensive possibilities for studying CTCs in different types of tumors in a cost-effective and straightforward way, there are still limitations and obstacles to consider. Several things, like the binding of NP probes and aggregation, could change how accurate and consistent NP-based detections are, which would make them less reliable and repeatable. Furthermore, the majority of NP-based assays are primarily developed for academic research purposes, and their practical application in clinical settings is still limited. Furthermore, NPs have the potential to exhibit toxicity.

Although CTC capture and downstream analysis appear to be possible and have therapeutic significance, the majority of the procedures described are not currently being used on a regular basis. In order to successfully implement CTC technologies in a clinical context, it is necessary to address the existing restrictions. Getting a better understanding of epitope expression and plasticity is part of this. We also need to deal with problems like cell loss due to changes in size and shape, low purity of CTCs, device blockages, the need for a lot of blood, the time-consuming nature of the process, and the difficulty of automating it. Further difficulties pertain to enhancing functional assays, such as developing more effective culture techniques and CTC-derived xenografts. Also, molecular analysis needs to be thoroughly checked to make sure it works. This includes taking into account random changes, limited sequencing coverage, bias in amplification, high error rates, and different bioinformatics methods (92). Successfully addressing these obstacles has the potential to elevate CTCs to a prominent position in personalized medicine, serving as minimally intrusive yet highly informative biomarker sources.

A method called multi-targeted magnetic capsules (TMCs) was created by Ma et al. It combines natural immune recognition with tumor-specific ligand targeting. The goal of this method is to capture rare CTCs. In general, the fluorescence-visible magnetic capsules (MCs) were created by using an ultra-sonication assembly method. This involved combining fluorescently labeled thiolated hyaluronan (RhB-HA-SH) and hydrophobic Fe3O4 NPs. Superparamagnetic Fe3O4 particles could not move during this process because RhB-HA-SH cross-linked them with it through the oxidation of the thiol groups. FA and an anti-EpCAM antibody were added to the hyaluronan MCs to change them. These modified capsules were able to trap and isolate over 88% of uncommon MCF-7 cells in mimic biological materials within a 15-min incubation period. Furthermore, these individual cells exhibited favorable capabilities for both cell division and movement, which greatly aids in doing additional research on CTCs. In addition, these TMCs were effectively utilized for the collection and identification of a small number of CTCs in 1.5-ml samples of peripheral blood from patients with cancer. This suggests that they have the potential to be a viable option for the capture and identification of CTCs using a straightforward and rapid magnetic manipulation technique (93).

## 6 Membrane biomimetic nanoparticles

**Figure 6:**
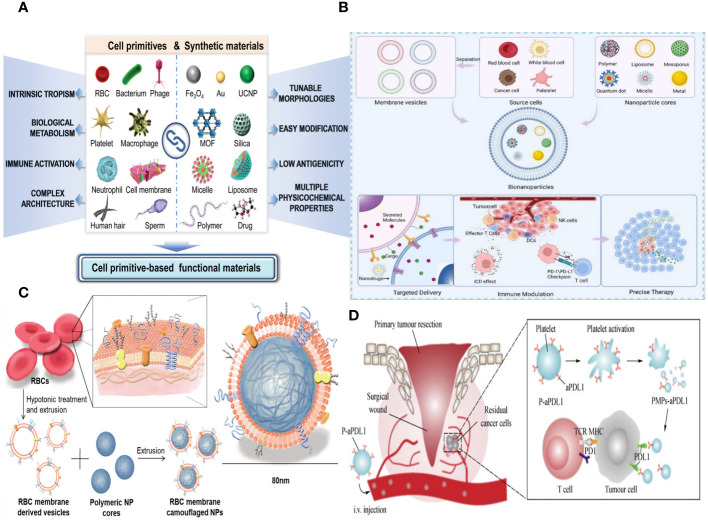
**(A)** Schematic of the different forms of cell primitives and synthetic materials, respectively, and their potential as building blocks to fabricate cell primitive-based functional materials as newly emerging therapeutic formulations. **(B)** A schematic diagram of the preparation of membrane-coated biomimetic nanoparticles and their functions. **(C)** RBC membrane–coated nanoparticles. Cell membrane can be derived from RBCs using hypotonic treatment. **(D)** Schematic illustration of anti–PD-L1 delivery to primary-tumor resection sites by platelets, where TCR is T-cell receptor, and MHC is the major histocompatibility complex.

The cell membrane is a lipid-bilayer structure that encompasses the periphery of the cell. It is composed of proteins and polysaccharide components (94). The primary roles of cell membranes encompass barrier function, material exchange, and information exchange. The cell membrane serves as a protective barrier between the cell and its surroundings, shielding the cell from potential harm and safeguarding the organelles within it (34). The cell membrane facilitates the transfer of chemicals between the cell and its surrounding environment. The cell membrane exhibits selective permeability, allowing small molecules to freely traverse, whereas larger biological molecules, such as proteins, require endocytosis for cellular entry. Furthermore, cell membranes are equipped with several ion pumps that are crucial for upholding cellular osmotic pressure and internal homeostasis. Cell membranes play a crucial role in facilitating both the transfer of substances and the transmission of information between the cell and its surroundings (95). The cell membrane contains a variety of membrane receptors that are crucial for intracellular signal transmission and are necessary for controlling cell proliferation, migration, apoptosis, metabolism, and other biological activities (**Figure 6A**) (96).

It is common and easy to get biomimetic cell membrane nanotechnology, which means that biomimetic NPs have good chances of being used in immunotherapy to fight cancer. Cell membranes, including those from red cells, leukocytes, platelets, cancer cells, and hybrids, can serve as biomimetic nanotechnology materials (**Figure 6B**). Bio-nanoparticles are able to demonstrate distinct cell–like functionalities due to the inherent characteristics and features of cell membranes. Researchers can select appropriate membrane materials based on the distinct characteristics of each membrane type, in accordance with specific requirements. Enclosing NPs with EMs has the potential to enhance the NPs’ long-term cycling capacity and reduce their elimination by the immune system (20). Leukocyte membranes trigger the immune system and specifically attack tumors (97). Bionic devices utilizing the platelet cell membrane can be employed for the treatment of tumor metastasis (98). NPs, which are covered with the membranes of tumor cells, exhibit homologous targeting toward tumors (99). On the other hand, the hybrid membrane preserves the characteristics of the original cell membrane and possesses several roles (100). Moreover, it is feasible to enhance the functionality of cell membranes, thereby increasing the adaptability of NPs. In this article, we provide a comprehensive analysis of several cell membrane–coated technologies used in the context of cancer applications.

### 6.1 Erythrocyte-derived membrane biomimetic nanoparticles

An erythrocyte is a highly prevalent and abundant blood cell that has a longer duration of circulation in the blood and is responsible for transporting oxygen to different organs and tissues (101). Erythrocytes have been thoroughly studied for their possible application in medication administration due to their capacity to circulate within the bloodstream and readily detach from it (102). The incorporation of the EM can greatly enhance some attributes of nanocarriers. The employment of electromagnetic fields can greatly enhance some attributes of nanocarriers. Initially, it serves to safeguard the functionality of the enclosed material, allowing for a prolonged and regulated lifespan while also preventing its elimination by the immune system. A lot of CD47 is found on the outside of the EM. CD47 is a receptor that binds to the signal-regulatory protein alpha that is found on macrophages. This interaction stops the immune system from clearing NPs, allowing them to circulate in the bloodstream for a longer period of time and preventing immune reactions (22). Furthermore, erythrocytes play a major role in the transportation of oxygen due to their abundance of hemoglobin, which has a strong affinity for oxygen molecules and facilitates their delivery to various tissues in the body (103). Furthermore, it is noteworthy that EM can undergo complete degradation within a living organism without generating any harmful byproducts, hence showcasing its favorable characteristics of being biocompatible and biodegradable for *in vivo* uses. The use of EM-encapsulated nanocarriers can also effectively lower toxicity and improve stability by lowering the formation of aggregates. Hence, nanocarriers that are coated with EM material offer numerous benefits in the field of nanodrug delivery (**Figure 6C**).

EM biomimetic NPs can protect different membrane proteins, glycans, CD47, and acidic sialyl groups, which stops macrophages from taking them up in the bloodstream without a specific purpose (104). Modifying the surface of EM with targeting groups may compromise its immune evasion capability. However, the surface of targeting biomimetic NPs can maintain their structure and proteins, optimizing the biomimetic drug delivery system’s targeting capabilities and minimizing side effects on healthy organs. Combining EM and NPs can create a biomimetic delivery system that makes NPs more biocompatible, stable, and able to target cells, allowing them to evade the immune system.

### 6.2 Platelet-derived membrane biomimetic nanoparticles

Platelets are anucleate hematopoietic cells originating from the bone marrow (105). Platelets possess a more intricate structure than erythrocytes, featuring several surface receptors that facilitate their interaction with the cellular milieu (106). Fully understanding how platelets, the TME, and the immune system are connected should lead to the creation of drug delivery systems based on platelets and effective methods for targeting platelets in the treatment of cancer (107).

Platelet membranes (PMs), like EM, have the ability to transmit signals to macrophages that discourage them from engulfing them. This mechanism allows nanocarriers coated with PMs to remain in circulation for longer periods and avoid being detected and eliminated by the immune system (108). Furthermore, the presence of P-selectin (P-sel) on PMs facilitates a targeted interaction with CD44 receptors on tumor cells, hence enhancing the accumulation of drugs in the tumor tissues (**Figure 6D**) (109). Because CD47 is on the PMs, PM-encapsulated liposomes were better able to avoid being destroyed by the immune system.

PM-covered drug delivery devices are good at going after living cells and could be useful for treating cancer because they do not get eaten by phagocytes. However, their implementation in clinical settings remains challenging due to inadequate technology for large-scale manufacturing, supply, and storage and the lack of understanding of the relationship between platelets and tumor tissues, necessitating further research.

Researchers have developed a biomimetic single-atom nanozyme system to improve the effectiveness of nanocatalytic tumor treatment (NCT) through self-enhancement. They synthesized copper Single-atom nanozymes (SAZs) with exceptional POD-like activity using a high-temperature carbonization method. The SAZs were then merged with PM vesicles to form a proton pump inhibitor (PPI). PPIs controlled hydrogen ions, glutathione (GSH), and H_2_O_2_ levels in tumor cells, enhancing the catalytic capacity of SAZs and facilitating self-enhanced NCT. PPS demonstrated a tumor suppression rate of over 90% in a living organism and effectively restricted GSH production within cells, incorporating glutamine metabolism therapy and NCT into a groundbreaking approach. This method offers a fresh and effective strategy for multimodal tumor therapy (110).

### 6.3 Immune cell–derived membrane biomimetic nanoparticles

**Figure 7:**
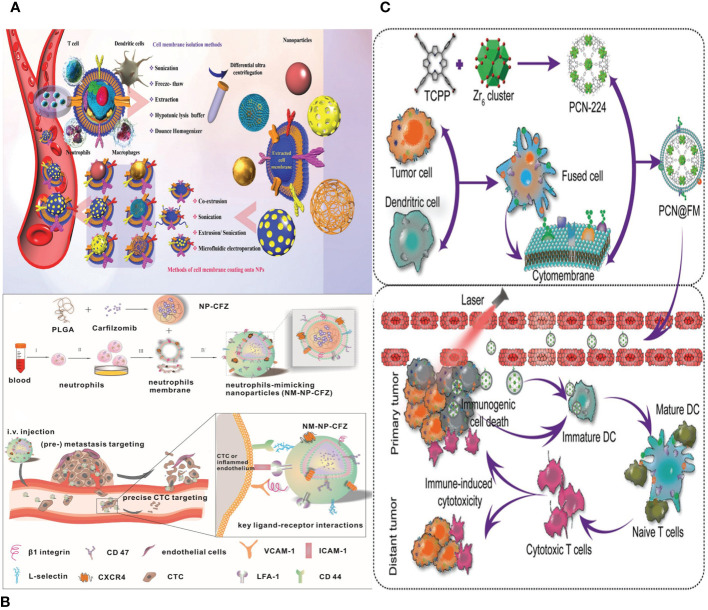
**(A)** Different source immune cells and various types of NPs formed via camouflaging different cell membranes. First, immune cell membranes are isolated from blood or their other sources and then extruded to obtain membrane vesicles. Finally, the vesicles fuse with core NPs to form membrane-camouflaged NPs. **(B)** Schematic illustration of neutrophils-mimicking nanoparticles loaded with carfilzomib (NM-NP-CFZ) that selectively deplete CTC and their site of colonization. **(C)** Schematic illustration of PCN@FM for combined tumor therapy.

Immune cells, which originate from hematopoietic stem cells in the bone marrow, are the biggest blood cells. They consist mainly of macrophages, neutrophils (NEs), DCs, NK cells, and lymphocytes (111). The membrane composition of immune cells is more intricate than that of RBCs and platelets, and it includes distinct proteins and glycans that are absent in other cell membranes. Thus, immune cells have the ability to generate active immunological responses in order to eradicate inflammation and suppress the formation of tumors (112). Lipid-hybrid immune cell–derived bionic functional materials show significant potential in the realm of targeted therapies (**Figure 7A**).

#### 6.3.1 Macrophage-derived membrane biomimetic nanoparticles

Macrophages are a subset of innate immune cells that originate from monocytes. They consist mostly of two types: M1, which stimulates inflammation, and M2, which suppresses inflammation. The predominant macrophages in the TME are primarily of the M2 phenotype. Research has indicated a strong correlation between tumor growth, movement, reappearance, and other biological processes in TAMs. TAMs possess the ability to specifically identify tumor cells and exosomes (113). Therefore, the utilization of biomimetic NPs created through the coating of macrophage membranes serves as an efficient approach for targeting tumors.

Macrophages are powerful immune system cells that can react vigorously to various mediators, strengthening the immune system’s defense against tumors. They are ideal for disguising NPs designed to target tumors due to their ability to specifically attack tumor cells. A study created bionic nanocarriers by enveloping synthetic Fe_3_O_4_ NPs with macrophage membranes, mimicking their behavior (114). This process extends the nanocarriers’ circulation duration, allowing them to selectively locate tumor cells and prolong their circulation duration. This technique is crucial in tumor prevention and treatment, particularly metastasis.

Xin Huang et al. conducted a study where they synthesized Fe_3_O_4_ biomimetic NPs coated with a macrophage membrane (Fe_3_O_4_@M). The purpose of this work was to evaluate the imaging effect of these NPs on the early lesions of atherosclerosis, specifically foam cells. The findings indicated that the Fe_3_O_4_@M particles exhibit a spherical shape with an average diameter of around 300 nm. The T1 and T2 relaxation measurements indicated that the ratio of r2 to r1 was 26.09. The protein content constituted around 27% of the overall weight in Fe_3_O_4_@M, and the Fe_3_O_4_@M NPs demonstrated significant biosafety. Later tests showed that Fe_3_O_4_@M can effectively target early atherosclerotic lesions by specifically binding to integrin α4β1 and VCAM-1 (115).

#### 6.3.2 Neutrophil-derived membrane biomimetic nanoparticles

Neutrophils are the predominant leukocytes in the blood and play a crucial role in the innate immune system. They are able to enter tissues outside of blood vessels through a process called diapedesis (116). The damaged tissues, pathogens, and other inflammatory cells present at the site all produce various chemokines when there is tissue damage, inflammation, or infection. Neutrophils possess advanced membrane receptors that detect these chemokines, guiding them toward the inflamed region. Neutrophil membranes show a lot of promise for being used to make biomimetic NPs that carry nanodrugs effectively and precisely.

NEs can be directed specifically to cancer cells that have spread or areas that are inflamed by biomimetic functional materials that contain NE components (112). NPs enclosed in a membrane have been created to specifically target tumors. The nanocarriers have the ability to impede the growth and spread of tumors both in laboratory settings and in living organisms, as well as trigger programmed cell death in early-stage tumor cells and CTCs (97). Furthermore, NE membrane–bound liposomes have been employed for precise medication administration in the context of pancreatic cancer (**Figure 7B**) (117).

#### 6.3.3 Dendritic cell–derived membrane biomimetic nanoparticles

DCs, which act as guardians of the immune system, play a crucial role in initiating and controlling adaptive immunological responses (118). Antigen-presenting cells (APCs) are highly efficient in recognizing pathogenesis-associated molecular patterns and danger-associated molecular patterns via pattern recognition receptors, making them the most functioning among all APCs. DCs initiate the activation of the immune system by internalizing and processing antigens and subsequently presenting antigen-specific information to T cells (119).

NPs with a DC membrane around them can activate APCs, move peptide antigens, and make it easier for memory T cells to respond to antigens. Multiple investigations have shown that DC membranes containing tumor antigens can specifically target lymph nodes to promote immune activation (**Figure 7C**) (120). Combining DC membrane–based immunotherapy with other treatments can generate synergistic anti-tumor benefits. In summary, DC membrane–encapsulated NPs have the ability to specifically target lymph nodes and stimulate T-cell responses.

Lipid-hybrid DC–derived biomimetic functional materials are promising nanocarriers for treating tumor spread through the lymphatic system. These membrane vesicles can guide drugs toward lymph nodes by activating APCs and transporting peptide antigens. When combined with immune checkpoint inhibitors, these materials can lead to heightened and long-lasting anti-tumor immunity, making them a promising option for treating tumors.

#### 6.3.4 T-cell–derived membrane biomimetic nanoparticles

T cells have the ability to directly eliminate tumor and virus-infected cells and also play a crucial role in coordinating the overall process of elimination (121). They begin in the bone marrow and undergo differentiation into several types of effector cells as they undergo proliferation and differentiation. Additionally, they undergo a transformation into memory cells, which actively contribute to a subsequent immune response against foreign substances.

T cells can potentially act as a membrane reservoir for biomimetic nanotechnology. T cells originate from pluripotent stem cells in the bone marrow and are responsible for cellular immunity. To activate cellular immunity, two main processes work. The first is T cells binding specifically to target cells, which destroys the target cell membrane and kills the target cell directly. The second is lymphokines being released, which help the immune system work better and more broadly. T-cell membranes possess many markers, including the T-cell antigen receptor (TCR), major histocompatibility complex (HLA) antigens, and interleukin receptors. These markers play a crucial role in triggering immunological responses (122).

#### 6.3.5 NK cell–derived membrane biomimetic nanoparticles

NK cells play a crucial role in the innate immune response (123). Equipped with membrane receptors, these cells have the ability to identify anomalous cells, such as tumor cells (124). They play a big part in controlling the immune system by killing target cells directly, releasing cytokines, and helping APCs mature (125).

**Figure 8:**
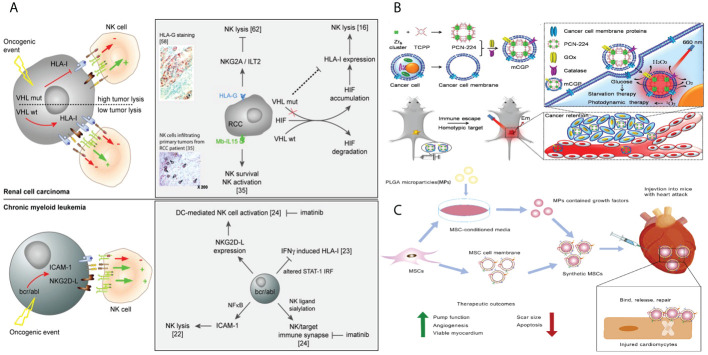
**(A)** Tumors parameters implicated in the activation of NK cells. **(B)** Schematic illustration of the cancer cell membrane camouflaged cascade bioreactor for cancer targeting starvation therapy and PDT. **(C)** Mesenchymal stem cell membrane–coated growth-factor-loaded nanoparticles for tissue repair. Synthetics MSCs release loaded growth factors to promote tissue repair through cell proliferation, angiogenesis, and remuscularization.

The utilization of NK cell–based immunotherapy shows significant potential for its implementation in clinical settings. The present focus of research is on investigating the recruitment and infiltration of NK cells in tumors with the aim of enhancing treatment outcomes (126). The proposed solutions aim to augment the direct cytotoxicity of NK cells by promoting their concentration, specifically at the tumor location. Due to their inherent capacity to selectively attack tumor cells, particularly those exhibiting stem cell properties, researchers have explored the possibility of using NK cell membrane–encapsulated NPs for precise tumor targeting (**Figure 8A**) (127). The tumor-targeting ability of NK cells is likely due to the action of certain proteins on their cell membrane.

### 6.4 Tumor cell–derived membrane biomimetic nanoparticles

The membranes of tumor cells are obtained from tissues and cells taken from the patient and can be reproduced indefinitely in a laboratory setting. The material becomes easily accessible, and the bionic nanocoating derived from these membranes demonstrates the characteristics of a tumor cell. Metastasis is the merging of cancer cells to modify and restructure the surrounding environment of the tumor. The process of aggregation and adhesion is said to rely on the presence of surface-adhesion molecules such as N-cadherin, galectin-3, and EpCAM on the membranes of tumor cells (128). By using the inherent targeted adhesion property of these molecules, nanomaterials can be precisely and efficiently directed toward tumor cells.

Tumor cells can be cultivated *in vitro*, exhibiting limitless value-added characteristics. Tumor immunotherapy can utilize the cell membrane of cancer cells as a source for membrane-coated biomimetic nanotechnology (129). Tumor cells possess numerous distinctive characteristics, including the ability of their membrane coats to evade the immune system and selectively attack similar cells, enabling them to prolong their cell cycle. In recent years, there has been extensive research on biomimetic tumor nanovaccines in the field of tumor immunotherapy. These nanovaccines have the ability to stimulate various immune responses against tumor antigens (130). The advantage of using biomimetic nanotechnology is that it allows NPs to retain tumor-specific antigens (TSAs), enhancing their ability to target tumors and deliver drugs. This enhances the strategy for tumor immunotherapy. So, it is worth looking into the cell membranes of cancer cells as a possible source of membranes for nanobiomimetic technologies with membrane coatings (**Figure 8B**) (131).

The study by Xiao et al. involved the creation of ZIF-8 NPs, which were ferrous ion–doped and engineered to resemble the membrane of cancer cells. These NPs were mixed with dihydroartemisinin (DHA) to create a treatment for cancer that specifically targets cancer cells while minimizing damage and adverse effects. The CDZs, which are biomimetic nanomaterials, possess remarkable homologous targeting capability and can specifically accumulate in tumor tissues. Within an acidic TME, the breakdown of materials might result in the release of ferrous ions and DHA. DHA, a traditional Chinese medication, synergizes with ferrous ions to generate a potent anti-tumor effect. Tumor growth in human liver cancer models was inhibited by around 90.8%. Also, the nanomaterial does not seem to be harmful or toxic to living things, and it is a powerful and safe way to treat tumors that has a lot of clinical benefits (132).

### 6.5 Mesenchymal stem cell–derived membrane biomimetic nanoparticles

Stem cells have been thoroughly investigated as a means of delivering genes, particularly for the purpose of cancer treatment. The formation of cancer stroma is comparable to the process of wound healing. Signaling molecules released by cancer cells help stem cells, especially mesenchymal stem cells (MSCs), to grow and become more numerous so that tissues can be formed (133). The combination of genetic engineering and the ability of stem cells to specifically target cancers allows for the expression of therapeutic genes that encode anti-tumor proteins, such as interferons and interleukins, within the tumor cells.

MSCs are versatile stem cells found in numerous tissues that have the ability to develop into different types of cells, such as osteoblasts, adipocytes, chondrocytes, and myofibroblasts. They are commonly employed in the fields of wound healing, chondrogenesis, and nerve regeneration (134). Due to their innate ability to specifically target tumors, MSCs can be utilized for the delivery of therapeutic medicines to tumor locations. Nevertheless, it is crucial to conduct thorough investigations to determine the exact function of MSCs in the formation of tumors in order to evaluate any potential hazards that they may provide, such as the emergence of metastasis (134). When comparing MSCs to MSCMs, it is pointed out that MSCMs provide a safer method for delivering drugs. MSCMs possess the inherent targeting capacity of MSCs and exhibit minimal immunogenicity. MSCM-encapsulated NPs have garnered significant attention due to their innate capacity to target tumors (135). MSC-derived nanostructures are being recognized as highly effective carriers for drug delivery due to their inherent characteristics, including their strong attraction to tumors, prolonged presence in the bloodstream, and minimal immune response (**Figure 8C**) (136).

### 6.6 Hybrid membrane biomimetic nanoparticles

**Figure 9:**
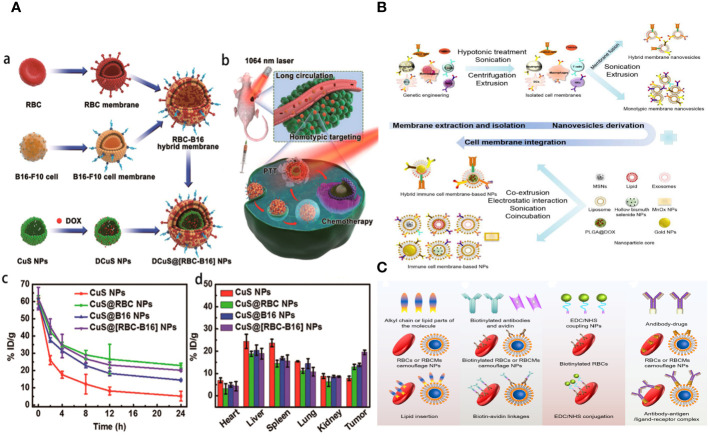
**(A)** Hybrid RBC-cancer cell membrane–coated hollow copper sulfide NPs for PTT. **(B)** Steps to synthesize biomimetic immune cell membrane–based nanoplatforms. **(C)** A schematic diagram of the surface modification methods of RBCMs-based nanomedicine.

Hybrid cell membranes (HMs) are designed to combine the properties of many types of cell membranes and enhance their practical functionality (137). In general, the structure of HM can be classified into two distinct components. One aspect is the inclusion of a payload that carries out precise and relevant tasks, such as delivering small molecular chemotherapeutic medicines or supplying photosensitizers for phototherapy (38). In this review, we concentrate on the second aspect, which involves equipping NPs with a hybrid membrane coating that allows them to effectively interact with their surroundings. This is achieved by improving their ability to target specific areas, minimizing their interactions with abundant proteins and cellular components that are not relevant, and enhancing their specific biological functions. The references for further reading on this topic are (29). In addition, hybrid membranes possess a minimum of two distinct biological functionalities. One feature involves the ability to target specific entities, while the other encompasses inherent characteristics conferred by the membranes of the originating cells (38). The targeting capability primarily involves the use of cancer cell membranes and DC membranes for homologous targeting of tumor sites (99). Additionally, PLT membranes are used for targeting specific tumors, whereas stem cell membranes enhance their ability to target tumors (136). Furthermore, PLT membranes and WBC membranes are employed for targeting CTCs (138). The second category of biological function primarily involves the extended circulation of RBCMs and PLT membranes; the targeted attachment to damaged blood vessels by PLT membranes; the evasion of the immune system by WBC membranes and PLT membranes; the neutralization and absorption of toxins by RBCMs and macrophage membranes; and the activation of the immune response by bacterial outer membranes, cancer cell membranes, and immune cell membranes (139). By utilizing several membranes, HM can optimize their functionality in diverse biomedical applications (**Figure 9A**).

## 7 Preparation methods, coating, and characterizations

The process of making biomimetic NPs for coating cell membranes mostly includes these four steps: (i) separating and getting the cell membranes; (ii) making very small cores at the nanoscale level; (iii) covering these nanocores with membranes; and (iv) validation techniques for cell membrane–coated NPs (140).

### 7.1 Isolation and extraction of cell membranes

The process of obtaining natural cell membranes involves cell lysis, the removal of cellular components, and further acquisition and purification. The key stage involves dissolving cells in a low osmotic pressure buffer, subjecting them to ultrasonic, homogenization, and repeated freezing and thawing (38). Cell destruction can be categorized into two types: chemical and mild lysis methods, and more rigorous techniques like mechanical disruption, ultrasound, stress homogenization, and pipetting. Techniques for isolating the cell membrane include ultrasonic treatment, freezing or thawing, extrusion, double homogenization, and a hypotonic dissolving buffer (**Figure 9B**) (112).

### 7.2 Fabrication of the nanocores

The inner nanocores play a vital role in the biomimetic nanoplatform, as they finally exert their influence at the desired locations. Size, shape, and surface charge of preformulated NPs are factors that affect the effectiveness of the final membrane cloaking (141). The ratio of nanocores to membrane materials determines the membrane coverage of NPs. Specifically, the NP cores must possess a negative charge in order to effectively coat the membrane (142). For instance, polylactide-co-glycolide (PLGA) with a terminal carboxyl group is preferred due to its negatively charged surface, which can repel the similarly charged outer layer of the cell membrane. This ensures the creation of structures with the correct orientation. Their preparation relies on traditional techniques, like emulsion solvent evaporation, nanoprecipitation, self-assembly procedures, or the membrane hydration method (143). They can be made of a lot of different types of NPs, such as PLGA, liposomes, nanogels, and inorganic materials like gold NPs, melanin NPs, SiO_2_, and Fe_3_O_4_ (139).

### 7.3 Wrapping of nanocores by membrane

Several techniques were devised to apply immune cell membranes to NPs. Co-extrusion (using mechanical force), extrusion, freezing and thawing, sonication (using ultrasonic energy), microfluidic electroporation (144), and stirring (using ultrasonic energy/endocytosis and exocytosis) are some of the most common methods used. One way to make NPs blend in with macrophage membranes is to co-extrude them with vesicles and to interact them with macrophage membranes (38). The process of membrane coating involves the utilization of the partially stable characteristics of the bare NP core and vesicles formed from cell membranes. These traits, along with the uneven distribution of charges on the biomembrane, allow for the formation of a dynamic core-shell structure with a specific membrane rotation direction to the right (29).

### 7.4 Improvement of targeting delivery by surface modification

Biomimetic NPs based on RBCMs can decrease the clearance of the RES and extend bloodstream circulation (125). However, these NPs may not deliver drugs specifically to tumor cells. To enhance the delivery efficacy of RBCMs-NCs, surface modification with ligands specific to tumor cell receptors is anticipated. Traditional chemical reactions face challenges in modifying the surface without altering protein structure. Consequently, sophisticated methods have been developed to enhance the surface properties of RBCMs, including incorporating the lipid phase, connecting biotin-avidin, conjugating 1-ethyl-3-(3-dimethylaminopropyl) carbodiimide/N-hydroxysuccinimide (EDC/NHS), and forming complex antibody–antigen/ligand–receptor interactions (145). This study opens up a novel approach to treating tumors and has accelerated the development of dually modified biomimetic NCs (**Figure 9C**).

A highly efficient CM biomimetic graphene nanodecoy was created through deliberate surface engineering, utilizing polyethylene glycol (PEG) to modify magnetic graphene oxide (MGO) and enhance its stability in a physiological solution. This modification aims to improve the screening effectiveness for active components of traditional Chinese medicine (TCM). By employing this approach, the synthesized PEGylated MGO (PMGO) demonstrated a remarkable stability for a minimum duration of 10 days, hence enhancing the efficiency of the CM coating. Utilizing the natural affinity of the HeLa cell membrane (HM) to bind with certain ligands, the HM-camouflaged PMGO demonstrated a satisfactory adsorption capacity of 116.2 mg/g and exhibited selectivity. Three possible active components, namely, byakangelicol, imperatorin, and isoimperatorin, were identified from *Angelica dahurica*. The potential antiproliferative activity of these components was confirmed through pharmacological investigations. The findings showed that purposeful surface engineering could be a way to make useful CM biomimetic nanomaterials. This will help find more active ingredients in TCM (146).

### 7.5 Verification methods for cell membrane–coated NPs

The final stage in the manufacturing of membrane-based NPs involves examining their unique attributes. The main parts of the analyses are looking at the structure, making sure membrane proteins are present, using fluorescent colocalization microscopy, and using ultraviolet-visible absorption spectroscopy (137).

The electron density, permeability, surface charge, and protein composition of cell membranes can be used to differentiate them from core NPs. These characteristics can be utilized to ascertain whether the membranes have been incorporated into the NPs (112). Transmission electron microscopy is used to observe NP morphology (137). In addition, dynamic light scattering is utilized to analyze the zeta potential and size distribution of NPs, with the coated NPs showing an increase in size compared to the uncoated ones (95). Additionally, fluorescence microscopy is used to check for colocalization and find out how two molecules are related in terms of where they are in the plasma membrane and where they are distributed within the nucleus (147).

## 8 Biomedical applications

NPs are widely employed in both diagnostic and therapeutic contexts. They demonstrate significant promise in various medical applications, including chemotherapy, PTT, detection of CTCs, radiotherapy (RT), diagnostic imaging, drug delivery, PDT, nucleic acid delivery, implantable devices, atherosclerosis therapy, heart repair, cancer vaccination, immunotherapy, tissue engineering, and HIV therapy (148). Upon entering the body, NPs encounter several obstacles. These include being quickly recognized as a foreign substance, which triggers immune responses; breaking down quickly and being removed from the bloodstream; not being able to work well with biological systems; being more toxic; and being absorbed quickly by the RES. In order to achieve successful drug delivery, it is crucial for NPs to possess long-lasting stability in the bloodstream, evade clearance by the mononuclear phagocyte system (MPS) and RES, accumulate inside the TME, penetrate the TME or tumor interstitial fluid, reach the desired location, and effectively interact with the target cells (38). Various synthetic and non-synthetic carriers have been suggested, but, nowadays, the focus is primarily on utilizing live cells and cell derivatives in modern applications. Therefore, researchers are enthusiastic about designing and developing NPs that have enhanced cellular characteristics. This is accomplished by adorning NPs with the source cell membrane, which enhances targeted interaction with the surroundings, overcomes bio-adhesion in the bloodstream, and provides biocompatibility, extended circulation time, and preferential accumulation in the TME (38, 112).

### 8.1 Drug delivery

**Figure 10:**
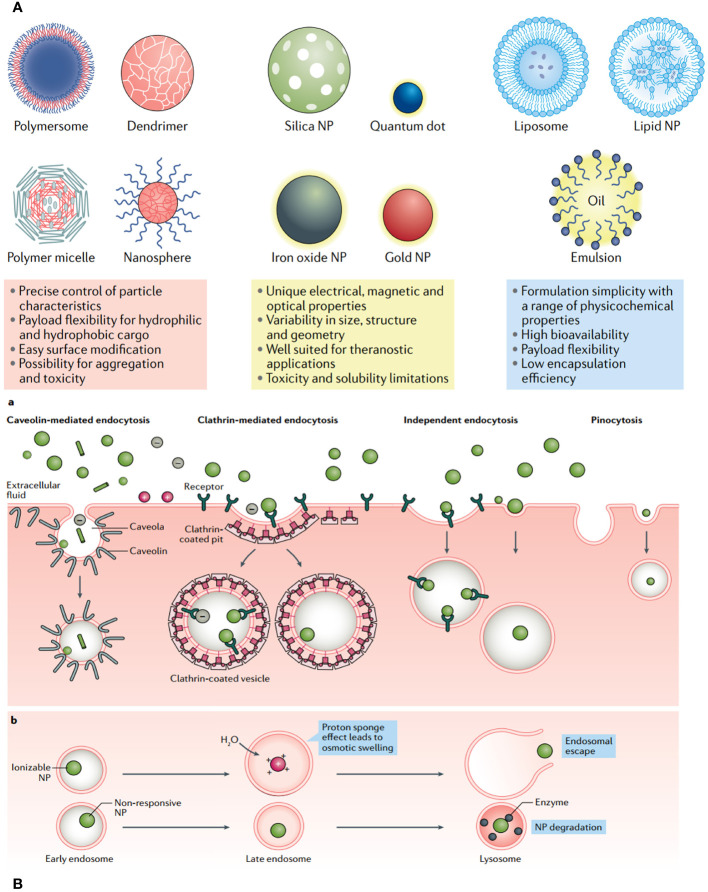
**(A)** Each class of nanoparticle (NP) features multiple subclasses, with some of the most common highlighted here. Each class has numerous broad advantages and disadvantages regarding cargo, delivery and patient response. **(B)** Common uptake pathways that ultimately determine NP fate within a cell.

Progress in drug delivery systems is being facilitated by advancements in diverse biomaterials (149). The desirable attributes of efficient drug delivery systems include (i) exceptional stability and prolonged systemic retention duration; (ii) the capability to surpass biological barriers; and (iii) enhanced active tumor targeting and regulated elease (150). NP-based drug delivery systems (NDDSs), such as polymeric NPs, liposomes, micelles, dendrimers, gold NPs, and carbon nanotubes, have become significant due to their distinct and adjustable physicochemical characteristics, such as shape, size, surface charge, ligand modification, and controlled drug release (**Figure 10**) (151). Passive NDDSs, which involve the accumulation of NPs in tumor tissue through improved penetration and retention, are classified as first-generation DDS (139). The active targeting NDDS is referred to as the advanced version of NDDS. It has the ability to specifically attach to target cells by utilizing specific ligands such as antibodies, peptides, aptamers, FA, integrin, and growth factors (7). Nevertheless, active targeting of functionalized NDDS encounters many obstacles, such as: The problems with this treatment are (i) that it can hurt areas that are not supposed to be treated, (ii) that it can interact with healthy tissues and biological parts (especially proteins) that are not specific to the target, and (iii) that immune cells can get rid of it.

The cell membrane–based carrier system uses its natural physiological properties to get the following benefits over NPs that are not designed with membranes: Combining active tropism, minimal immunogenicity, and physiological barrier permeability seems to be a better way to deal with the problems listed above.

RBCMs are often used as natural carriers due to their ability to effectively bind with other membranes and control the circulation duration of HM@BNPs. PMs possess considerable potential to confer NPs with versatile characteristics, such as the ability to evade the immune system and selectively adhere to damaged blood vessels or specific tumors. This has a substantial therapeutic influence on the field of nanomedicine (139). The cancer cell membrane has been extensively utilized in membrane-coated NP technology due to the presence of many surface indicators, such as CD44, glycoproteins, integrins, selectins, and cadherins (17). The leukocyte membrane has been utilized as a natural biomimetic coating material to evade capture by the immune system and target inflammation through targeted receptor-ligand binding (152). Research has demonstrated that macrophages play a role in the initial spread of cancer and therefore have a substantial impact on the long-term development of metastasis in cancer progression (153). To summarize, dual-fused membrane-based nanoagents offer a significant benefit compared to existing invasive therapies.

Chen et al. developed an anticancer NP disguised as a PM using indocyanine green, poly(d,l-lactide-co-glycolide), and PM. The study assessed the impact of irradiation pretreatment on the nanomaterial’s tumor targeting and efficacy. The PINPs@PM nanomaterial, made from indocyanine green, poly(d,l-lactide-co-glycolide), and PM, was exposed to 4-Gy X-ray radiation, resulting in an increase in G2/M phase cells and Caveolin-1 content. This led to hyperthermia and reactive oxygen species, which were harmful to the cytoplasmic lysosome, and cell death. The anticancer activity of PINPs at PM was enhanced *in vitro* by irradiation pretreatment. *In vivo* studies on mice showed that irradiation significantly increased the tumor-targeting ability of INPs at PM. This approach combines irradiation pretreatment and PM camouflage to transport anticancer NPs, potentially playing a crucial role in targeted tumor therapy (154).

### 8.2 Phototherapy

**Figure 11:**
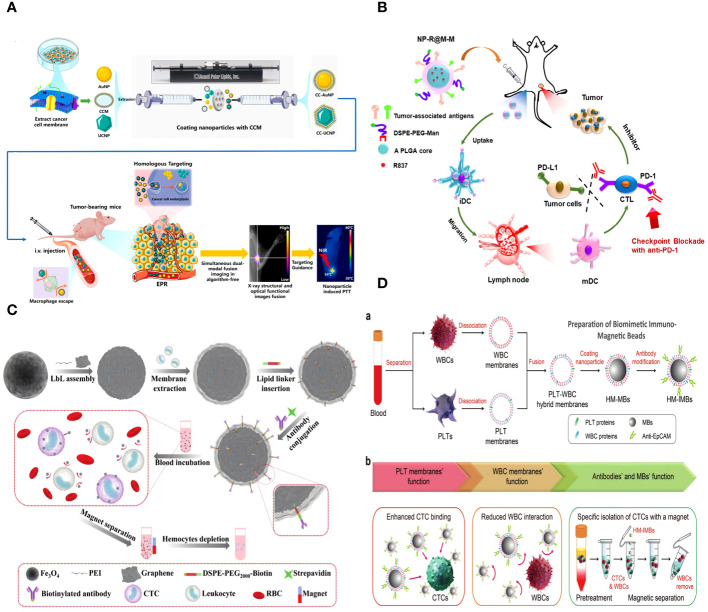
**(A)** Schematic of the synthesis and application of cancer cell membrane–coated gold nanoparticles (CC-AuNPs) and cancer cell membrane–coated upconversion nanoparticles (CC-UCNPs) combined with a novel simultaneous dual-modal imaging platform for guided photothermal therapy (PTT) for cancer. **(B)** Schematic illustration to show the structure of tumor cell membrane coated, R873 loaded, and mannose modified PLGA NPs (NP-R@M-M), and their functions to induce anti-tumor immunity as a nanovaccine. **(C)** Fabrication process of leukocyte-mimicking immunomagnetic nanoplatform and general CTCs isolation process in blood samples. **(D)** Schematic of the preparation of HM-IMBs for high-performance isolation of CTCs.

Phototherapy includes noninvasive methods such as PDT and PTT. PDT and PTT have demonstrated desirable advantages in cancer treatment, such as exceptional selectivity, precise control over the timing and location of light exposure to tumor tissues, the absence of inherent or acquired resistance mechanisms, and little harm to the body as a whole (155). Phototherapeutic chemicals are supplied either intravenously or through local injections, depending on the disease models. Nevertheless, typical phototherapeutic agents encounter significant constraints such as bio-adhesion and opsonization by serum proteins in the bloodstream, prompt identification and elimination by immunological sentinels and the MPS, and limited accumulation in targeted sites (156). There is hope that cell membrane camouflaging can help solve these problems because it has many surface proteins and biological properties that make it less likely to trigger immune responses, improve blood flow, and allow for targeted delivery. Additionally, this approach preserves the photophysical properties of phototherapeutic NPs. The extended lifespan of RBCMs is crucial for enhancing the effectiveness of photo-based applications (157). The adhesive capabilities of platelets can enhance the uneven distribution of phototherapeutic chemicals in phototherapy. This is because the thermal damage generated by post-injury feedback triggers the recruitment of new platelets, which, in turn, enhances the targeting ability of the delivery system (158). Leukocyte membrane–encapsulated NPs have been shown to be very appropriate particles for phototherapy. They have the potential to enhance the biocompatibility and targeting ability of PTA or photosensitizers when administered systemically (159). Stem cells and cancer cell membrane–coated nanocarriers have been developed for phototherapy to significantly improve the effectiveness of tumor treatment (**Figure 11A**). This is because they have the ability to specifically target tumor cells and accumulate at the tumor (131). Combining the aforementioned cell membranes can enhance the therapeutic efficacy of phototherapy (160).

Chen and his team have developed PM-coated CuP NPs (PCs) that can specifically target and deliver cancer to the cancer site. This approach overcomes heat resistance by tumor cells, enabling effective and gentle PTT. CuP uses near-infrared II (NIR-II) laser irradiation to transform light energy into heat energy and catalyzes the production of highly poisonous ·OH, effectively eliminating tumor cells. The use of PM coating enhances the circulation of PC NPs within living organisms and their concentration at specific tumor locations. CuP, which acts like peroxidase, lowers the expression of Heat Shock Protein (HSP) 90. This makes cells less resistant to heat, which makes CuP-mediated mild PTT work better. Both *in vivo* and *in vitro* experiments show that the combination of PC with the NIR-II laser effectively kills tumors and is biologically safe. This presents further possibilities for the effective implementation of PTT in clinical settings (161).

### 8.3 Tumor vaccine

Tumor vaccines have emerged as a prominent area of research in recent years. Tumor vaccines aim to administer diverse tumor antigens, such as altered tumor cells, tumor-associated proteins or peptides, and genes encoding tumor antigens (162). Tumor vaccines have the ability to counteract the immunosuppressive effects of tumors, increase the ability to provoke an immune response, stimulate the patient’s own immune system, and trigger both cellular and humoral immune reactions to manage or eradicate tumors. Recent research has utilized cancer cell membrane vesicles (CMVs) to enclose immune-adjuvant NPs, resulting in a very efficient approach for anticancer vaccination (**Figure 11B**) (163). Mature APCs can target and treat specific antigens found on the membranes of cancer cells, which, in turn, activate cytotoxic T lymphocytes to initiate immune responses against the tumor. However, the immunological activation of CMV alone in APCs is restricted, which is unsatisfactory. Essentially, the bionic approach of both eukaryotic and prokaryotic organisms can successfully trigger a targeted immune response against tumors. This presents a novel concept for developing combination vaccines against cancer. The coalescence of DCs and cancer cells facilitates the incorporation of a wider range of tumor antigens into vaccines, hence optimizing the immune response that specifically targets the tumor.

### 8.4 Immune therapy

A common strategy for anti-tumor immune therapy is to supply the immune system with tumor antigen materials, which can be processed and presented by the body’s immune stem (164). However, the immune responses to tumors are frequently diminished due to the presence of tumor heterogeneity and immmunosuppression (21). Identifying appropriate tumor antigens for immunotherapy is challenging due to the heterogeneity of TSAs and tumor-associated antigens, as well as the unclear understanding of some of their immune processes (165). Only the presence of cancer cell membranes is insufficient to trigger an immune response. The fusion of membranes has the ability to induce the maturation of immature DCs and initiate a series of immune responses. Additionally, the cancer cell membrane has a uniform ability to specifically target melanoma cells.

### 8.5 Detoxification

Pore-forming toxins often target human normal cells, including RBCs, leading to significant hemolysis reactions in the majority of cases (15). As a result, scientists created nanosponges coated with RBCMs, which have the ability to neutralize toxins and protect healthy RBCs (166). Macrophage-mimicking NPs are more suitable for catching toxins compared to RBCM-coated NPs because they possess the ability to capture gram-positive bacteria produced from the membrane (167).

## 9 Cell membrane biomimetic nanoparticles for CTCs

Metastasis is responsible for more than 90% of cancer-related deaths in clinical settings (168). The spread, seeding, and colonization of CTCs are the primary factors that lead to the development of distant metastasis. CTCs have become a powerful diagnostic tool and therapeutic target for the prevention of metastasis. The manipulation of CTCs has been challenging until the emergence of CTC-targeted nanomedicine. An anti-EpCAM antibody has been designed to specifically target CTCs and disrupt their connection with endothelial cells. Nevertheless, the malignant heterogeneity can pose significant challenges to the precision and effectiveness of these nanomedicines that target EpCAM. In order to tackle the issue, researchers have devised cell membrane “camouflaged” methods to specifically target CTCs and their micro-thrombi (97).

### 9.1 Capture and detection of CTCs

The CTCs found in the peripheral blood are strongly associated with the progression, spread, and reappearance of solid tumors. Hence, it is imperative to advance novel biological technologies for the effective separation and detection of CTCs in blood. The utilization of nanotechnology in liquid biopsy has demonstrated its significant benefits due to its high surface-to-volume ratio, which facilitates the effective capture and enrichment of biomarkers. For instance, CTCs, which are strongly associated with tumor size, advancement, spread to other parts of the body, recurrence, and prognosis, have become more common in monitoring the evolution of tumors and showing the effectiveness of treatments (169). The immune magnetic bead (IMB) enrichment method utilizes magnetic beads (MBs) as a medium for separation and antibodies attached to the surface of MBs for targeted binding to CTCs. This strategy has been demonstrated to be highly significant in the early detection and isolation of tumor cells. Despite its potential, IMB enrichment currently encounters certain constraints. The initial challenge lies in the constrained cellular recuperation rate, which stands at around 40% in the artificially contaminated blood samples. In addition, the background of approximately RBCs and WBCs per milliliter of whole blood remains, which leads to a reduced purity after separation and has an impact on the subsequent bioassay (170). Cellular membranes can serve as an innovative platform for the surface modification of nanostructures and protect against unwanted complex elements like regular blood cells and cell debris.

Zhou et al. combined graphene nanosheets with magnetic NPs (MNs) using the LbL assembly method. They then incubated them with leukocytes to create membrane-coated biomimetic magnetic NPs (BMNs). Lastly, they put a biotinylated lipid linker between the CTC-targeting antibodies and the BMNs (**Figure 11C**) (171). The biomimetic immunomagnetic NPs (BIMNs) that were created were very good at finding leukocytes (capture efficiency, >85.0%), absorbing anti-leukocytes more efficiently (purity, >94.0%), and being very sensitive (as few as three CTCs in 1 mL of blood) in blood samples that did not have many CTCs. Furthermore, the utilization of BIMNs on peripheral blood samples obtained from patients with cancer showcased the excellent accuracy and consistency of our platform (average relative standard deviation, 8.7 ± 5.6%).

Rao et al. combined PLT and WBC membranes to create PLT-WBC hybrid membranes. These hybrid membranes were subsequently applied to MBs and modified with specific antibodies on their surface (**Figure 11D**) (172). The PLT-WBC hybrid membrane–coated immunomagnetic beads (HM-IMBs) obtained possess an improved capacity to attach to cancer cells inherited from PLTs while minimizing interactions with homologous WBCs. These HM-IMBs are subsequently employed for the extremely effective and highly specific extraction of CTCs. Through the utilization of spiked blood samples, it has been discovered that HM-IMBs exhibit an enhanced cell separation efficiency of 91.77% compared to the 66.68% efficiency of commercial IMBs. Additionally, the cell purity of HM-IMBs has been improved to 96.98% from 66.53%. In addition, the HM-IMBs enable the successful identification of very pure CTCs in 19 out of 20 clinical blood samples obtained from patients with breast cancer.

**Figure 12:**
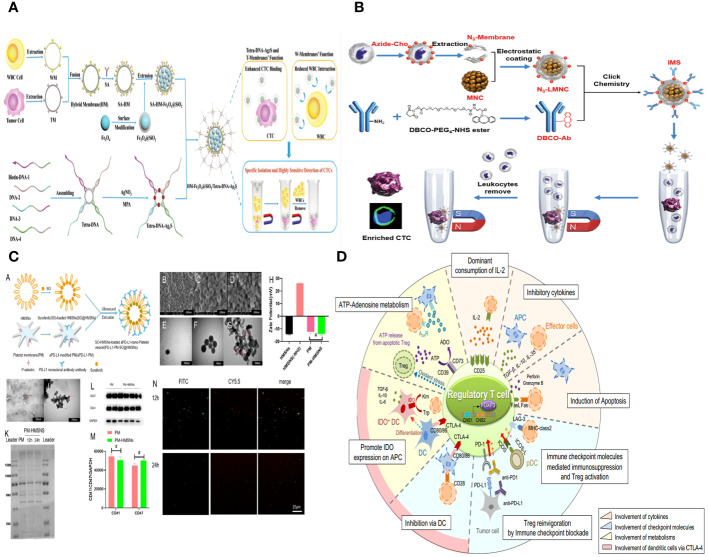
**(A)** Illustration of the preparation of HM-Fe_3_O_4_@SiO_2_/Tetra-DNA-Ag2S nanoplatform for the efficient isolation and detection of rare CTCs. **(B)** Construction of IMS and the procedure of CTC enrichment. **(C)** Targeted co-delivery of PD-L1 monoclonal antibody and sorafenib to circulating tumor cells via platelet-functionalized nanocarriers. **(D)** Suppressive mechanism of regulatory T (Treg) cells.

Ding et al. made a nanoplatform by connecting Ag2S nanodots to magnetic NPs that were coated on the membrane of the MCF-7-RAW264.7 cancer cell. This was done to enhance the capture efficiency and detection sensitivity of CTCs, as demonstrated in their study (**Figure 12A**) (173). As anticipated, the utilization of WBC membrane–coated materials enhanced the efficacy of isolating CTCs by minimizing interference caused by similar leukocytes. Furthermore, NPs coated with tumor cell membranes have an exceptional ability to specifically target cells of the same type, thanks to their inherent self-recognition. Also, Ag2S nanodots (NDs) were created as aptamer-functional NDs that can attach to immune-magnetic NPs that have been changed with streptavidin (SA). It was easier to catch and identify CTCs with the new nanoplatform because it improved the interaction between cancer cells and CTCs while also reducing the effect of WBCs in the background. Remarkably, the average rates of retrieval for artificially introduced cells in treated blood and unprocessed blood were 96.24% and 90.25%, respectively, as observed in samples taken from human subjects. Overall, the hybrid membrane–coated Ag2S nanobioprobe has promising potential for clinical applications in detecting CTCs and diagnosing diseases.

By changing some ligands on the cell membrane, bioorthogonal reactions can make biomimetic systems better at targeting. Xie et al. developed a novel biomimetic immunomagnet (IMS) for the purpose of enriching CTCs by utilizing N3-modified leukocyte membranes (**Figure 12B**) (174). According to research (175), the absence of CTCs in the bloodstream makes CTC enrichment or targeting difficult. The researchers employed a bioorthogonal process to affix the EpCAM onto the surface of IMSs. EpCAM is a widely used cancer marker that is considered to be a highly effective antigen for detecting CTCs. IMSs specifically target CTCs that express EpCAM, leading to a substantial accumulation of IMSs on the surfaces of these CTCs. By employing a magnetic field and microfluidic technology, it is possible to extract over 90% of CTCs from whole blood within a time frame of 15 min. This biomimetic system addresses the issue of CTC enrichment, which is crucial for the detection and treatment of cancer. By conjugating DBCO-GRD to the surface of macrophages by a bioorthogonal process, magnetic NPs containing siRNA can be delivered to tumor cells that have an abundance of RGD receptors, facilitated by a magnetic field.

Zhu et al. conducted a study where they coated tumor-targeting chemicals FA and magnetic NPs (MNPs) onto the surface of RBCs using hydrophobic contact and chemical conjugation, respectively. The modified RBCs attached quickly to CTCs, and the resulting CTC-RBC conjugates were separated using a magnetic field. Following the application of RBC lysis buffer and subsequent centrifugation, CTCs were liberated and collected. The entire process lasted for just 3 h. The cell counting analysis revealed a capture rate over 90% and a purity of the collected CTCs exceeding 75%. The proposed technique outperformed MACS^®^ beads in terms of capture efficiency (80%) and purity (20%) under identical conditions. The collected CTCs were able to be cultivated and grown in a laboratory setting (176).

Hu and colleagues have developed a novel strategy to prevent damage from foreign material and achieve maximum efficiency. They use molecules that specifically target tumor cells to bind them to RBCs that are similar in structure. This creates noticeable differences in optical properties, such as size and refractive index, between the RBCs conjugated with CTCs (CC-RBCs) and other blood cells. Subsequently, the altered CTCs can be accurately distinguished using laser light within an optofluidic system. Results from experiments show that red blood cells can be used to change CTCs, and, then, they can be extracted from blood with a high level of purity (above 92%) and a high rate of retrieval (above 90%). Throughout the entire process, it has been demonstrated that CTCs maintain both membrane and functional integrity. When homologous RBC binding is combined with an optofluidic system, it creates a useful tool for finding cancer early and keeping an eye on how well treatment is working. This approach demonstrates excellent performance in the non-invasive and accurate extraction of CTCs, hence displaying significant potential (177).

Xie et al. present a composite material consisting of a magnetic Fe_3_O_4_ core enclosed in a MIL-100 shell (MMs). This material is capable of responding to changes in pH and may be modified with anti-EpCAM on the surface of MIL-100, resulting in anti-EpCAM-MMs. There is no need for any additional conditions once anti-EpCAM-MMs have captured the cells. However, due to the acidic environment in the cell culture process, MIL-100 can undergo automatic disintegration, resulting in the release of the cells. This self-release strategy has the potential to enhance cell viability while also streamlining the release procedure and conserving human and material resources. Furthermore, they integrated the clinical diagnosis of patient cases with DNA sequencing and advanced RNA sequencing technology, aiming to achieve precision medicine for future patients (178).

Zhang et al. developed a platform based on a smart DNAzyme probe. Efficient capture and selective release of different types of CTCs were achieved by utilizing a combination of numerous targeting aptamers and multiple metal-ion-responsive DNAzymes. The DNAzyme Sgc8c needs Cu^2+^ to work, so it may be able to catch CCRF-CEM cells on the substrate. The TD05 aptamer, which has a DNAzyme that depends on Mg^2+^, can also capture Ramos cells on the substrate. It is possible to selectively separate CCRF-CEM cells or Ramos cells from the substrate by adding Cu^2+^ or Mg^2+^. Their capture/release approach holds promise for further research on the molecular characterization of CTCs post-release and offers significant potential for cancer diagnosis and personalized treatment (179).

### 9.2 Eliminate CTCs

Within the bloodstream, the survival of CTCs is affected by immunological pressures, interactions with blood cells, and the stress caused by fluid movement. However, it is not feasible to identify and eradicate CTCs that are found in the peripheral blood due to their scarcity, with only around one in a million leukocytes (and/or a billion erythrocytes) in circulation. The movement of CTCs in the bloodstream is comparable to that of leukocytes (180). The distinctive characteristics of leukocytes, such as their resemblance to tumor cells in terms of their ability to travel through the bloodstream, adhere to the walls of blood vessels, and migrate toward tumor and inflammatory sites, make them a valuable tool for effective cancer therapy.

Neutrophils, being part of the innate immune system, are among the initial cells to combat pathogens. Given this information, neutrophils have been proposed as promising carriers for delivering drugs to treat malignancies. Granulocyte colony-stimulating factor mediates a pathway that attracts both neutrophils and CTCs after the establishment of a specific area. Neutrophils can use molecules like lymphocyte function-associated antigen-1 (LFA-1), L-selectin, and β1-integrin to help CTCs find the premetastatic niche and capture it. These molecules bind to their respective ligands, intercellular adhesion molecule-1 (ICAM-1), CD44, and Vascular Cell Adhesion Molecule-1 (VCAM-1) (97). Additionally, neutrophils can trap CTCs via neutrophil extracellular traps. Neutrophil-mimicking NPs, specifically neutrophil membrane–coated NPs (NM-NPs), have been created and studied in cancer treatment experiments. These NPs possess similar characteristics to neutrophils and have been shown to inhibit tumor growth and metastasis, as well as induce apoptosis in premetastatic tumor masses and CTCs in both laboratory settings and living organisms. The presence of CTCs in premetastatic niches has been demonstrated to be elevated following exposure to NM-NPs (97). Additional advanced methods, such as nanovehicles coated with neutrophil and macrophage membranes (NM-NVs and MM-NVs, respectively), have demonstrated efficacy in inhibiting tumor growth and migration. They achieve this by eliminating CTCs and impeding the formation of metastatic nodules. Hence, these nanocarrier systems possess the capacity to serve as agents for combating tumors and inhibiting metastasis.

CTCs have the ability to bind to and stimulate platelets, resulting in the formation of a microthrombus barrier. This barrier serves to prevent therapeutic drugs and immune cells from effectively targeting and eliminating the CTCs. The PM bionic drug delivery system possesses a robust capacity for immune evasion and can remain in circulation inside the bloodstream for an extended period of time. Inspired by the adhesion of activated platelets to CTC-related microthrombosis, Da et al. developed a preventative treatment for post-surgical tumor recurrence. They functionalized Sorafenib–loaded hollow mesoporous silicon particles with activated PM vesicles and coupled antibodies against Programmed death-ligand 1 (PD-L1) to the PM (**Figure 12C**) (181). It is easier for synthetic drug-loaded particles and immune checkpoint inhibitors to get to the cancer cell thrombus in the circulatory system with this biomimetic coating. It achieves this by leveraging the interaction between CTCs and platelets, ultimately leading to the eradication of tumor cells.

NK cells play a crucial role in monitoring the immune response against tumors and preventing the spread of cancer cells by identifying and eliminating them. CTCs can be influenced, either indirectly or directly, to regulate the spread of cancer. Platelets adhere to CTCs, providing a protective layer that shields them from both mechanical forces and NK cell–mediated immune responses. NK cells have an influence on cancer metastasis by employing diverse methods and engaging with CTCs via distinct ligands and receptors (**Figure 12D**) (182).

T cells display heightened expression of adhesion molecules on their cell surface in comparison to other immune cells. Multiple pathways can lead to T-cell dysfunction, which facilitates the spread of DTCs and/or CTCs, promoting metastasis. For instance, regulatory T cells (Tregs) have the ability to shield tumor cells from being recognized by the immune system, enabling their survival, growth, and acquisition of characteristics that facilitate the spread of cancer to other parts of the body. Tregs demonstrate immunosuppressive capabilities (182). Therefore, utilizing T-cell biomimetic delivery platforms that display tumor-specific T-cell receptors can serve as extremely precise medication carriers that target metastasis.

Jarvas and his colleagues describe the alteration of hemodialysis membranes that are sold in stores to specifically trap CTCs in the bloodstream. This is achieved by attaching human anti-EpCAM antibodies to the inner surface of the fibers. The text describes the necessary processes for adding the immuno-affinity feature to hemodialyzer cartridges in order to capture CTCs that are positive for EpCAM, which accounts for approximately 80% of cancer cell types. Outcome: By putting HCT116 cancer cells into both a buffer solution and whole blood before running them through the modified cartridge, it was possible to determine how well the suggested method captured cells. The approach’s cell clearance performance was quantitatively evaluated using flow cytometry. The proposed modification does not have a substantial impact on the porosity structure of the hemodialysis membranes. It maintains its ability to remove cytokines and also addresses cachexia by removing CTCs simultaneously (183).

## 10 Prospects and challenges of clinical applications

### 10.1 Based on CTCs

In order to successfully integrate technologies into the therapeutic setting, it is necessary to acknowledge and overcome their inherent limits. To do this, we need to learn more about epitope expression and plasticity, as well as how to deal with problems like cell loss due to changes in size and shape, low purity of CTCs, device blockages, the need for a lot of blood, the time-consuming nature of the process, and the difficulty of automating it. Further obstacles pertain to enhancing functional assays, such as developing more effective culture techniques and CTC-derived xenografts. Also, molecular analysis needs to be thoroughly checked to make sure it works. This includes taking into account random changes, low sequencing coverage, amplification bias, high error rates, and different bioinformatics methods (183). Successfully addressing these problems could ultimately position CTCs as prominent contributors to personalized medicine, serving as minimally intrusive yet highly informative biomarker sources. Currently, there are still various obstacles that need to be overcome before liquid biopsy may be more commonly used in the detection and monitoring of malignancies.

#### 10.1.1 Maximizing CTC detection

The primary obstacle hindering the use of CTCs for early cancer detection is the limited presence of these cells in standard blood samples, which reduces their effectiveness in identifying cancer. Consequently, it is crucial to optimize the quantity of CTCs accessible for examination. Considering the naturally limited quantity of CTCs produced by early-stage malignancies, an apparent strategy is to enhance the detection technologies’ sensitivity. The task of capturing all CTCs has been challenging using epitope-dependent separation strategies. As a result, researchers have directed their efforts toward designing and implementing isolation methods that rely on cell size, density, or morphology to enhance the yield of CTCs (184). Each of these technologies has limits. For example, classic epitope–based isolation platforms may not be able to detect EpCAM-negative CTCs that are undergoing EMT. Similarly, devices that select CTCs based on cell size may not be able to capture small CTCs. Therefore, employing a combination of various selection approaches could enhance the efficiency of collecting CTCs for research purposes. However, it is important to note that while intricate sequential selection procedures are likely to enhance the purity of CTCs, they may also result in a higher loss of cells throughout each processing stage. Ongoing validation of CTC detection technologies will be crucial in finally gaining approval for these systems for the early identification of cancer. Another constraint is the present absence of the ability to replicate liquid biopsy experiments due to the absence of uniformity across various laboratory operations. Subsequent research should assess the technical resilience and replicability of suggested biomarkers within and among laboratories by employing a standardized protocol. Given the present level and rate of technological advancement and our comprehension of the biological and physical characteristics of tumor cells, it is likely that *in vivo* detection of CTCs for early cancer diagnosis will become possible in the near future. To attain this ambitious objective, it will be necessary to foster multidisciplinary collaboration among cancer researchers, physicists, bioengineers, and doctors, both in university and industry contexts.

#### 10.1.2 Molecular characterization of CTCs

Moreover, if we can better separate and find CTCs, then doing more molecular research on these cells might help us tell the difference between slow-growing cancers that are clinically significant and those that are not. There is a paucity of thorough characterization of CTCs. Because there is only a limited amount of genomic DNA, RNA, and protein in CTCs, it is hard to study their genome, transcriptome, epigenome, and proteome. However, the advancing technology of single-cell sequencing has lately been beneficial for genome and transcriptome studies of CTCs. In contrast, proteome studies of CTCs remain challenging due to the limited technologies available for examining proteomes at the single-cell level. Still, studying the CTC proteome is important because it can help us understand the biological properties of CTCs and find CTC-specific membrane proteins that could make CTC detection more accurate. In relation to the solid TME, the blood microenvironment surrounding CTCs also has a substantial impact on the ability of the tumor to survive and invade. Nevertheless, our understanding of the fundamental mechanisms responsible for the survival of CTCs remains restricted. This is due to the intricate nature of the process, which encompasses not only shear forces and fluid mechanics but also soluble substances and extracellular vesicles associated with the tumor.

#### 10.1.3 Clinical applications in cancers

Before these techniques can be used in clinical settings, more research needs to be done to find out how they work, what tissues the parts of a liquid biopsy come from, and what biological role they play. In the context of early-stage malignancies, this is especially crucial but encounters significant obstacles in terms of sensitivity and specificity. This issue could be resolved by conducting a comparative analysis of CTCs alongside other circulating analytes such as circulating tumor DNA and exosomes. This would enhance both the accuracy of identifying positive and negative cases as well as the ability to compare and integrate liquid biopsies with standard of care (SOC) methods. CTCs have been confirmed as autonomous prognostic indicators, thus expanding the conventional TNM system. In addition to counting, we anticipate that CTCs will demonstrate their capacity as prognostic indicators for categorizing patients according to the identification of therapeutic targets or resistance mechanisms, including ongoing monitoring. We need to do time- and intervention-controlled clinical trials to find out if treatment decisions based on CTC readouts will be better or more effective than treatment decisions based on regular tissue biopsies. More research needs to be done to see if liquid biopsy can be used to predict how different types of cancer will respond to treatment and to see how well different liquid biopsy analytes work. Additional endeavors to establish uniform analysis platforms and integrate liquid biopsies as a complementary biomarker in extensive pharmacological studies are also justified.

### 10.2 Based on cell membrane biomimetic nanoparticles

NP biomimetic approaches have been well-designed for drug delivery systems with immune escape, active targeting, longer blood circulation time, excellent tumor therapy outcomes, and minimal systemic toxicity compared with traditional drug delivery systems. However, the application and investigation of biomimetic NPs are still at an infant stage. There are various challenges and problems that need to be solved, including the source of the cell membrane, the fabrication process of biomimetic NPs, and the safety, biocompatibility, and targeting ability of these biomimetic NPs in tumor therapy.

#### 10.2.1 Application prospects of cell membrane biomimetic nanoparticle

Functional materials produced from cells exhibit excellent biocompatibility and possess particular bioactivities. These biological activities are essential in the identification, therapy, and prediction of tumors. NP-hybrid cell–derived biomimetic functional materials have been employed for tumor treatment due to the subsequent attributes: 1) Tumor targeting: biomimetic functional materials obtained from cellular components such as tumors and immune cells possess inherent tumor-targeting properties. These materials can significantly boost the ability to target tumors, improve the effectiveness of anti-tumor treatments, and minimize negative side effects. Biomimetic materials facilitate precise medication distribution for tumor treatment and enhance the efficacy of CTC detection. 2) Immune evasion: The immune system has the ability to detect and remove NPs that are transporting medicines. CD47 is present on the membranes of red blood cells, platelets, and tumor cells. It can attach to SIRPα on phagocytes, which prevents phagocytosis and lowers the chance of NPs being cleared by the MPS while circulating. 3) Inducing anti-tumor immunity: Tumor vaccines can stimulate APCs by targeting specific antigens found on tumor cells. This triggers an immune response that selectively targets and eliminates tumor cells. This approach is known for its high specificity and few side effects (118). Tumor cell–derived biomimetic functional materials can serve as tumor vaccines, stimulating anti-tumor immune responses and leading to targeted tumor cell destruction (112). OMVs can act as immunological adjuvants, augmenting their anticancer effects (185). 4) Enhanced drug-carrying capacity: While cell-derived biomimetic functional materials possess inherent abilities such as tumor targeting, immunological evasion, and activation of anti-tumor immune responses, their limited drug-loading capacity hinders their use in drug delivery techniques. The integration of NPs with cell-derived biomimetic functional materials enhances the drug-loading capacity, leading to improved efficacy in targeting tumors, recognizing the immune system, and triggering anti-tumor immune responses.

#### 10.2.2 Challenges of cell membrane biomimetic nanoparticle

Putting together NPs and biomimetic functional materials derived from cells can use their individual strengths to make the anti-tumor effect stronger. Through additional investigation into bionanomaterials, the utilization of NP-hybrid cell–derived biomimetic functional materials will have a significant impact on the treatment of tumors. However, the following problems need to be solved in order for NP-hybrid cell–derived biomimetic functional materials to move forward: 1) Various cell membranes originate from different sources, such as erythrocytes and platelets, which are derived from the body’s prevalent and easily accessible blood cells (161). However, the fabrication method for tumor cell membranes and immune cell membranes is laborious and involves the cultivation and multiplication of large samples *in vitro* (186). When biomimetic NPs are introduced into the body, they can trigger a strong immune response. Hence, it is important to take into account the development and fabrication of biomimetic NPs using cell membranes that are similar to those found in the patients’ affected areas. 2) Uneven or partial distribution of NPs can trigger the body’s immune response to destroy the biomimetic NP system. Hence, preserving the structural integrity of the cell membrane is a crucial concern that must be addressed during the fusion and extraction procedures (137). Currently, the most commonly used method for extracting cells while preserving the functionality and integrity of the cell membrane is by repeating the freeze-thaw procedure and using hypotonic therapy (23). Nevertheless, many existing treatments are currently in a state of little or no progress during the initial research phase, necessitating further time and steps to enhance them until they are suitable for clinical use. Prior to clinical application, the most crucial considerations are safety and biocompatibility. At present, cell membrane biomimetic technology demonstrates superior biocompatibility and targeting capability in comparison to other conventionally modified approaches. Nevertheless, these trials are now limited to basic and preliminary research conducted on mice. Further *in vivo* experiments and detailed information are necessary to provide a comprehensive understanding.

### 10.3 Applications of cell membrane biomimetic nanoparticles in CTCs

CTCs can offer insights into genomic alterations in tumor sites and investigate the metastatic process of cancers, encompassing aspects such as cell shape, migratory capacity, and sensitivity to drug-induced stimulation. They play a crucial role in the early detection of tumors, monitoring diseases, evaluating the effectiveness of drugs, and tailoring treatment to individuals. Yet, the retrieval of CTCs from blood presents considerable obstacles due to their exceedingly low prevalence and the indiscriminate attachment of other blood constituents. Also, most modern release strategies use techniques that are too close to cells and can damage their function, which makes capture less effective, capture less pure, and cell activity lower. The use of nanostructured substrates can greatly improve the effectiveness of capturing CTCs. However, they often clump together, have a weak magnetic response, are not very good at recognizing antibodies, and tend to adsorb leukocytes without being specific. These characteristics are not favorable for downstream investigation of CTCs. Biomimetic nanocarriers with membranes have many of the same properties as the original cells, such as being highly biocompatible, causing little or no immune response, staying in the body for a long time, being able to deliver drugs specifically, and being able to activate either innate or adaptive immunity. They have been effectively created in the areas of drug delivery, PTT, tumor vaccinations, and other related domains. Thus, in order to enhance the purity of captured CTCs, it is optimal to apply a coating of the cell membrane onto the surface of the capture substrate. By incorporating NPs that replicate cell membranes onto the capture substrate, several benefits are achieved. These include enhanced target recognition, improved protection of cells against external stimuli, and increased purity of captured cells. Moreover, the pliable interfaces of cell membranes can protect the functionality of CTCs during the processes of capture and release. Nevertheless, the utilization of cell membrane biomimetic NPs in CTCs remains infrequent and is still in its nascent phase of advancement. Currently, the cell membranes that have been extensively researched are those of WBCs and platelets. These membranes can be easily obtained in huge quantities and have the highest promise for clinical applications. Nevertheless, a substantial amount of research must be conducted to ensure the biosafety of the hybrid membranes. Concurrently, it is necessary to create practical and straightforward techniques for extracting and characterizing membrane biomimetic NPs produced from cells.

To ensure accurate results in clinical testing, it is crucial to have patient adherence and conduct thorough preclinical system surveys to eliminate any potential factors that could affect the outcomes (152). Despite the numerous obstacles that cell-based nanoplatforms for CTCs still encounter in their journey toward clinical use, it is indisputable that nanoplatforms have immense promise in the treatment of cancer metastasis. We are certain that cell-derived membrane biomimetic NPs hold significant promise for broader applications in the field of CTCs.

## 11 Conclusions and future perspectives

Significant advancements have already been made in comprehending the mechanisms underlying the initial spread and metastasis of cancer within this swiftly progressing domain. Microscopic cancer cells that have spread to other parts of the body can emerge at an early stage of tumor formation. There is increasing data suggesting that CTCs can be identified during the initial phases of aggressive cancer growth. Hence, CTCs possess immense promise for early cancer detection, facilitating the recognition of medically significant tumors while preventing the unnecessary diagnosis of non-aggressive conditions. The current difficulty lies in creating tools to reliably extract and examine these scarce but implicative cells. As technology continues to advance, especially in the area of detecting CTCs using non-invasive or minimally invasive methods to collect a large volume of blood at a convenient time, we anticipate that CTC analysis will revolutionize early cancer detection and significantly enhance outcomes for patients with cancer.

Recent studies indicate that there are several subgroups of CTCs inside individual patients, characterized by varying phenotypic makeup and metastatic capacities. It is crucial to conduct focused research to determine the characteristics and genetic makeup of CTCs, with particular attention to understanding their ability to spread and form metastases. The integration of genomic, epigenomic, transcriptomic, and proteomic methods has allowed for the characterization of individual CTCs. This has provided valuable insights into the heterogeneity within tumors and the reasons behind resistance to therapy. Utilizing single-cell multi-omics techniques can potentially track the development of CTCs and create a comprehensive catalogue of cancer progression, which encompasses numerous targets that can be therapeutically exploited. The profiling of biomarkers obtained from CTCs is more clinically significant than relying solely on current diagnostic and prognostic methods. The fact that these biomarkers are present in the bloodstream provides a significant benefit.

The utilization of a cell membrane biomimetic technique has significantly contributed to the field of tumor therapy. This article provides a concise overview of various types of biomimetic NPs that mimic the cell membranes of erythrocytes, cancer cells, platelets, and immune cells. These NPs are designed to enhance their blood circulation, evade the immune system, and target tumors, ultimately leading to more effective anti-tumor effects. However, there are some problems that need to be fixed before biomimetic NPs can be used in medicine. These include a complicated process for making them, problems with large-scale production, low yields, and storage issues. The key requirements for the successful clinical application of the cell membrane biomimetic method should include precise delivery targeting, exceptional anti-tumor activities, extended circulation time, low adverse effects, and favorable economic outcomes.

The cell membrane bionic nanomagnetic beads possess the ability to remain undetectable to other cells and evade non-specific attachment and engulfment by other components of the blood, hence minimizing interference in the sample. The bionic nanomagnetic beads possess a cell membrane that exhibits fluidity. This flexibility makes it possible for antibody clusters to form that bind target cells more strongly during the recognition process. The purpose of this bionic nanomagnetic bead is to tackle the three challenges currently encountered by CTCs: inadequate capture efficiency, insufficient capture purity, and diminished post-release activity. This technology can efficiently and accurately capture CTCs in a complex blood environment while also allowing for their non-destructive discharge. This technology possesses significant clinical utility. Using biomimetic NPs that look like cell membranes for CTCs is still rare and in its early stages of development. However, using cell membrane camouflage to change NPs is already seen as an interesting and possible method. Currently, significant endeavors are being made to prioritize metastasis in therapeutic approaches, such as the Metastasis Working Group (124). By effectively applying our understanding of metastasis biology and utilizing effective ways to capture CTCs, we may significantly enhance our efforts in clinical trial design and clinical-translational frameworks. In our vision of the future, CTCs could play a crucial role in categorizing patients and determining treatment decisions by promptly identifying aggressive tumor subclones. This is important for developing exceptionally potent cancer treatments. In conclusion, directing efforts toward CTCs and their clusters has the potential to halt the progression of metastasis and enhance the chances of survival. In order to ensure the future adoption and widespread acceptance of CTC technologies by physicians, patients, and healthcare governing bodies, it is imperative to acquire dependable clinical data that can be used to standardize and reproduce these technologies.

## Author contributions

YZ: Writing – review & editing, Writing – original draft. JW: Writing – review & editing, Visualization, Supervision.
